# Novel CB1-ligands maintain homeostasis of the endocannabinoid system in ω3- and ω6-long-chain-PUFA deficiency[S]

**DOI:** 10.1194/jlr.M094664

**Published:** 2019-06-05

**Authors:** Ina Hammels, Erika Binczek, Inga Schmidt-Soltau, Britta Jenke, Andreas Thomas, Matthias Vogel, Mario Thevis, Dilyana Filipova, Symeon Papadopoulos, Wilhelm Stoffel

**Affiliations:** Center of Molecular Medicine (CMMC), Laboratory of Molecular Neurosciences, Institute of Biochemistry,* University of Cologne, 50931 Cologne, Germany; Cluster of Excellence, Cellular Stress Response in Aging Related Diseases (CECAD)† University of Cologne, 50931 Cologne, Germany; Institute of Vegetative Physiology, Center of Physiology and Pathophysiology,** University of Cologne, 50931 Cologne, Germany; Institute of Biochemistry§ Deutsche Sporthochschule (DSHS) Cologne, 50933 Cologne, Germany

**Keywords:** fatty acid desaturase 2-deficient mouse model, arachidonic acid, diet effects, lipid metabolism, ω3 fatty acids, cannabinoid receptor 1, polyunsaturated fatty acid, 20:3^5,11,14^-endocannabinoids, endocannabinoid system-orexin-circuitry, surrogate cannabinoid receptor 1 ligands

## Abstract

Mammalian ω3- and ω6-PUFAs are synthesized from essential fatty acids (EFAs) or supplied by the diet. PUFAs are constitutive elements of membrane architecture and precursors of lipid signaling molecules. EFAs and long-chain (LC)-PUFAs are precursors in the synthesis of endocannabinoid ligands of G_i/o_ protein-coupled cannabinoid receptor (CB)1 and CB2 in the endocannabinoid system, which critically regulate energy homeostasis as the metabolic signaling system in hypothalamic neuronal circuits and behavioral parameters. We utilized the auxotrophic fatty acid desaturase 2-deficient (*fads2*^−/−^) mouse, deficient in LC-PUFA synthesis, to follow the age-dependent dynamics of the PUFA pattern in the CNS-phospholipidome in unbiased dietary studies of three cohorts on sustained LC-PUFA-free ω6-arachidonic acid- and DHA-supplemented diets and their impact on the precursor pool of CB1 ligands. We discovered the transformation of eicosa-all *cis*-5,11,14-trienoic acid, uncommon in mammalian lipidomes, into two novel endocannabinoids, 20:3^5,11,14^-ethanolamide and 2-20:3^5,11,14^-glycerol. Their function as ligands of CB1 has been characterized in HEK293 cells. Labeling experiments excluded Δ8-desaturase activity and proved the position specificity of FADS2. The *fads2*^−/−^ mutant might serve as an unbiased model in vivo in the development of novel CB1 agonists and antagonists.

Conservation of the multifaceted structures of ω3- and ω6-PUFAs during evolution, their high reactivity for multi-purpose biological activities, high energy-demanding biosynthesis, and tissue- and subcellular membrane-specific distribution put PUFAs down to essential entities for mammalian homeostasis. Numerous nutritional studies suggest essential physiological and pathogenetic implications of dietary ω3- and ω6-PUFA supply and their ratio in current Western diet in the development of metabolic, cardiovascular, and neurodegenerative disorders awaiting molecular understanding (1–4). Molecular insight into the majority of their pleiotropic structures and functions has remained elusive (5, 6).

PUFAs are essential constituents of membrane phospholipid bilayers and lipoproteins, and precursors of several lipid signaling molecules, including two endocannabinoid families, *N*-acyl-ethanolamides (NAEs) and 2-monoacyl­glycerols (2-MAGs). *N*-arachidonoyl-ethanolamide (AEA) and 2-arachidonoyl-glycerol (2-AG) are the most dominant ligands of cannabinoid receptor (CB)1 and CB2. The endocannabinoid system (ECS) (7–10) consists of endocannabinoids, receptors CB1 and CB2, and associated anabolic and catabolic enzymes (11–13), and modulates the orexinergic inputs into the neuronal network in selective regions of the CNS, sensing nutrient availability for maintaining body energy homeostasis (14). Unlike the intensively studied structure-function relationship of CB1 and CB2 in the ECS of the CNS [for review see (15)], the role of the dietary supply of PUFAs and their transformation to lipophilic CB1 ligands has remained elusive.

Biosynthesis from essential fatty acids (EFAs) and dietary supply maintain homeostasis of the cellular pool of long-chain (LC)-PUFAs in mammals. Δ6-Fatty acid desaturase 2 (FADS2) is the key enzyme in the transformation of EFAs ω6-18:2^9,12^ (linoleic acid) and ω3-18:3^9,12,15^ (α-linolenic acid) to ω3- and ω6-LC-PUFAs, of which ω6-20:4^5,8,11,14^ [arachidonic acid (AA)] and ω3-22:6^4,7,10,13,16,19^ (DHA) are the most abundant representatives. We generated the fatty acid desaturase 2-deficient (*fads2*^−/−^) mouse to unveil the enigmatic pleiotropic functions of individual ω3- and ω6-LC-PUFAs in controlled sustained dietary studies. The *fads2*^−/−^ mutant is an unbiased auxotrophic in vivo system (16, 17), which bypasses the caveats of numerous acute and long-term feeding experiments attempting to modulate the PUFA pattern. Cohorts of WT and *fads2*^−/−^ mice were maintained on three different diets: basic normal diet (nd) containing EFAs, nd supplemented with ω6-AA, and nd supplemented with ω3-DHA.

The ω3- and ω6-LC-PUFAs are absent in the lipidomes of peripheral tissues of adult nd-*fads2*^−/−^ mice and are stoichiometrically substituted by ω6-20:3^5,11,14^ (sciadonic acid). ω6-Sciadonic acid is synthesized on an aberrant pathway from ω6-linoleic acid (18). Diets supplemented with ω6-AA and ω3-DHA systemically suppressed ω6-sciadonic acid synthesis.

In this study, we utilized the auxotrophic *fads2*^−/−^ mouse mutant to explore the impact of *a*) EFAs in the nd-*fads2*^−/−^ mouse, *b*) ω6-AA in the AA-*fads2*^−/−^ mouse, and *c*) ω3-DHA in the DHA-*fads2*^−/−^ mouse on the PUFA pattern in diacylglycerol structures of their phospholipidome and the derived endocannabinoid metabolomes of brain and the resulting physiological states of the system.

## MATERIALS AND METHODS

### *Fads2*^−/−^ mice

The *fads2*^−/−^ mouse line was developed in this laboratory (16) and, after 10 backcrossings, maintained on a C57Bl/6N background. WT mice were obtained from heterozygous *fads2*^+/−^×*fads2*^+/−^ crossings. All mice were genotyped by PCR analysis of tail DNA. Cohorts of gender- and weight-matched WT and *fads2*^−/−^ mice, of ages indicated in the experiments, were used in this study. Animal protocols followed the principles and practices outlined in the National Institutes of Health *Guide for the Care and Use of Laboratory Animals*, and breeding and tests were done with permission of the local authorities (LANUV NRW). The animal studies reported in this work followed the ARRIVE-Guidelines (19). All animals were kept under specific pathogen-free conditions. The light/dark cycle was 12/12 h; the animals had free access to water and a regular diet (nd), ω6-20:4 (AA)-supplemented diet, or ω3-22:6 (DHA)-supplemented diet (Altromin diet #1310; Altromin, Lage, Germany). **Table 1** summarizes the GC/MS analysis of the fatty acid composition of the diets used for the transformation of the nd-*fads2*^−/−^ into the AA- and DHA-*fads2*^−/−^ mouse lines. Altromin diet #1310 was used as the basic diet and contains the two EFAs, 18:2 and α-18:3, to prohibit EFA deficiency. AA was supplemented as ARASCO and DHA as DHASCO triglycerides, with 50% 20:4 (AA) or 22:6 (DHA) as single LC-PUFAs, respectively, in the nd. Heterozygous foster mothers and colonies of +/+ and −/− mice were maintained on the respective diets throughout their lifetime.

**TABLE 1.** GC/MS analysis of the fatty acid composition of the diets used for the transformation of the nd-*fads2*^−/−^ mice into the AA- and DHA-*fads2*^−/−^ mouse lines

Fatty Acid	Diet		
	nd (%)	ω6-AA (%)	ω3-DHA (%)
16:0	26	13	12
18:0	3	2	2
18:1	34	24	24
18:2	34	55	53
18:3	3	3	3
ω6-20:4	—	3	—
ω3-22:6	—	—	6


### Lipidome analysis

Total lipids were extracted from brain or serum of WT and *fads2*^−/−^ male and female mice, homogenized in an Ultraturrax in 10 vol of chloroform/methanol (C/M) 2:1 (v/v) and re-extracted with C/M 1:1 (v/v) and C/M 1:2 (v/v) for 1 h each at 37°C under a stream of nitrogen. The combined extracts of total lipids were dissolved in C/M 2:1 (v/v), washed with 2 M KCl and water, and taken to dryness in a stream of nitrogen. Phospholipids were separated in the solvent system chloroform/ethanol/triethylamine/water 60/70/70/14 (v/v/v/v), using HPTLC plates (Merck, Germany). Bands were visualized by primuline fluorescence (0.2% in 80% acetone). Lipid bands were collected on fritted glass filters and eluted with C/M 2:1 (v/v) into Sovirel tubes and concentrated under N_2_ for analysis by MS. For GC/MS analysis, fatty acids were esterified with 5% HCl-methanol at 80°C for 1 h. One volume of water was added and fatty acid methyl esters (FAMEs) extracted with hexane and concentrated under nitrogen.

### Syntheses of [D6]- and [1-C^13^]-labeled eicosa-5,11,14-trienoic acids

[D6]- and [1-C^13^]-labeled eicosa-5,11,14-trienoic acids were synthesized following established procedures (20). 1-Chloro-nonadeca-4,10,13-triyne was used as substrate for deuteration and 1-chloro-nonadeca-4,10,13-triene for [1-C^13^] labeling 1-chloro-nonadeca-4,10,13-triyne, starting from 1,7-octadyne via 1-chloro-undeca-4,10-diyne. *Cis*-semi-hydrogenation with H_2_ or deuterium yielded unlabeled and [D6]1-chloro-nonadeca-4,10,13-triene chloride exchange with NaCN or Na^13^CN 1-cyano-nonadeca all *cis*-4,10,13-triene. Saponification of the nitrile yielded 20:35,11,14-methylester and alkaline hydrolysis the respective labeled and unlabeled 20:3^5,11,14^ acids. Intermediates and labeled and unlabeled end products were controlled by GC/MS and NMR.

### Synthesis of [D6]20:3^5,11,14^-EA and 2-d6-20:3^5,11,14^-G

Three hundred and twenty milligrams (1 mmol) of 20:3^5,11,14^ acid in 15 ml of ethyl acetate, 115 mg (1 mmol) of n-hydroxysuccinimide, and 210 mg (1 mmol) of dicyclohexylcarbodiimide in 5 ml of ethyl acetate were stirred overnight. Dicyclohexyl urea was removed by filtration or centrifugation and concentrated on a rotary still. The residue was dissolved in 10 ml of THF and 125 mg (1 mmol) of *N*,*N*-dimethylaminopyridine and 200 μl of ethanolamine or 200 μl of glycerol added and stirred at room temperature overnight. Completion of the reaction was assessed by HPTLC [solvent system: acetone/hexane 80/20 (v/v)]. Reaction products were extracted three times with methylene chloride, the combined extracts concentrated for purification by SiO_2_-chromatography: solvent, 5% acetone in hexane increasing up to 20%. Alternatively, the endocannabinoids were synthesized using the coupling reagent, 3-dimethylaminopropyl-3-ethylcarbodiimide, and *N*,*N*-dimethylaminopyridine.

Intermediates and labeled and unlabeled end products were controlled by GC/MS and NMR.

### Endocannabinoid analysis

Endocannabinoids were isolated following a modified previously described method (21). In brief, internal standards of D8-20:4 AEA, D8-20:4 AG, and D4-18:2 linoleoyl-ethanolamide (LEA) were supplied to brain and serum samples of WT and *fads2*^−/−^ female mice and extracted with acetone/PBS 3:1 in an Ultra-Turrax homogenizer. Samples were centrifuged (10,000 *g*, 10 min, 4°C). Supernatants were transferred to silanized tubes and evaporated to dryness under nitrogen. PBS (100 μl) and C/M (300 μl) 2:1 were added to the residue, vortexed for 15 s, and the two phases separated by centrifugation. The bottom phase was evaporated and reconstituted in 100 μl of methanol/water 3:1.

For calibration aliquots of 0, 10, 25, 50, 100, 250, 500, 750, 1,000, and 2,000 pg/μl of palmitoyl-ethanolamide (PEA), oleoyl-ethanolamide (OEA), LEA, AEA, 2-PG, 2-oleoyl-glycerol, 2-linoyl-glycerol, and 2-AG, 40 ng of D4-LEA, D8-AEA, and D8-2-AG and 10 ng of D4-PEA, D4-OEA, and D4-AEA in 2% BSA were used. Aliquots were treated as described for samples.

High resolution full scan MS measurements were accomplished using an Agilent Technologies (Waldbronn, Germany) HPLC coupled to an AB Sciex (Darmstadt, Germany) TripleTOF 5600 mass spectrometer. The system was equipped with a Thermo Fisher Scientific (Dreieich, Germany) Accucore C8 (2.6 μm particle size, 50 × 3 mm) analytical column, and mobile phases consisted of aqueous formic acid 0.2% (pH 2) (solvent A) and acetonitrile as organic modifier (solvent B) for optimal ESI conditions. Thereby, the gradient was held for 0.5 min and then decreased from 50% A to 0% A with 0.325 ml/min within 6 min. Afterwards, the column was washed for 1 min with 100% B. The subsequent re-equilibration time was 3 min. The mass spectrometer was calibrated frequently (after 10 injections) via the Duo Turbo-V-Ion source by a calibrant delivery system containing the manufacturer’s calibrants for positive ionization. The nitrogen for the ion source as well as collision gas supply was delivered by the nitrogen generator (CMC, Eschborn, Germany). Product ion experiments were acquired by isolating the respective [M+H]^+^ precursor ions in the quadrupole (unit resolution) and performing collision-induced fragmentation in the collision cell.

### Cell culture, transfection, and incubation experiments

WT and *fads2*^−/−^ mouse embryonal fibroblasts (MEFs) were prepared according to an established protocol (22). MEFs and HEK293 cells were grown in Dulbecco‘s modified Eagle’s medium (Seromed) supplemented with 10% fetal calf serum (Life Technologies, Inc.), 2 mmol glutamine, 1 mM sodium pyruvate (Biochrom), 100 units/ml penicillin, and 100 μg/ml streptomycin in a humidified incubator at 37°C in a 5% CO_2_ atmosphere.

HEK293 cells were mock-transfected with mammalian cell expression vector pRP[Exp]EGFP/Hygro-CAG>, fads2-transfected with pRP[Exp]EGFP/Hygro-CAG>mFads2, and *cb1*-transfected with pRP[Exp]EGFP/Hygro-CAG>mcb1, cloned by CyagenBiosciences, using a Bio-Rad Gene Pulser for electroporation (400 V, 250 μF). Cells were selected for 2 weeks using hygromycin-containing selection medium.

For incubation experiments, cells were starved for 6 h and incubated for 16 h in Dulbecco’s modified Eagle’s medium (Seromed) supplemented with 5% delipidated serum and 100 μM of [1-^13^C]ω6-18:2^9,12^ and/or [D6]ω6-20:3^5,11,14^ final concentration. Cell layers were washed and harvested for lipid analysis.

Changes in intracellular Ca^2+^ concentration [Ca^2+^]_i_ were measured using the Fluo-4AM (Thermo Fisher Scientific) calcium indicator as described in (23). Briefly, cells were loaded with 5 μM of Fluo-4AM dissolved in sterile imaging buffer [125 mM NaCl, 5 mM KCl, 2 mM CaCl_2_, 1.2 Mm MgSO_4_·7H_2_O, 25 mM HEPES (pH 7.4)] for 20 min at room temperature and subsequently washed with 2× 1 ml of imaging buffer. Each measurement consisted of a series of 500 images recorded at a rate of 0.254 s per frame (512 pixels^2^) for a total of 127 s via the FV-ASW 1.7 software on an Olympus FV1000 confocal microscope (488 nm excitation at 0.3% intensity, >505 nm emission). Stimulation of CB1 was measured by the fluorescence intensity evoked by Ca^2+^ release after ligand addition 5–10 s after the initiation of an image series recording.

Individual cells were marked as regions of interest (ROIs), and the average fluorescence was measured for each ROI. Background of each image was subtracted from each pixel of the average fluorescence (F) of a cell-free area. To account for the fluorescence of the expression marker, EGFP, a short series of 10 frames was taken immediately prior to Fluo-4AM loading and comparison of the average fluorescence in 10 ROIs before and after loading was used to calculate the increase in fluorescence caused by Fluo-4AM loading. For the analysis of CB1 agonist-induced Ca^2+^ transients, the fluorescent signals emanating from each of 10 ROIs (i.e., 10 cells) were evaluated for each image series via the formula ΔF/F_0_, where F_0_ is the average fluorescence intensity of the first five frames taken before ligand addition and ΔF is F − F_0_.

### Real-time PCR

RNA was isolated from WT and *fads2*^−/−^ male and female brains of littermates using Trizol (Invitrogen). Total RNA (10 μg) was reverse-transcribed using a Transcriptase kit (Life Technologies). Primer pairs used in quantitative PCR reactions are listed in **Table 2**. Hgprt was used as internal standard. Quantitative PCR reactions were performed with the ABI Prism 7900HT employing a 96-well format and Fast SYBR Green Master Mix (Applied Biosystems) following the manufacturer’s protocol. Data analysis was performed using the 2^−ΔΔCt^ method.

**TABLE 2.** Primer pairs used in quantitative PCR reactions

cb1 s	5′-agacctcctctacgtgggctcg-3′	
cb1 as	5′-gtacagcgatggcagctgctg-3′	
cb2 s	5′-gactctgggcctggtgctggctgtgctgct-3′	
cb2 as	5′-ctaggtggttttcacatcagcctctgtttc-3′	
faah s	5′-gattgagatgtatcgccagtc-3′	
faah as	5′-gaatgttgtcccacatatccc-3′	
trpv1 s	5′-ccagacagagaccctaactcc-3′	
trpv1 as	5′-cagctcctggcagttgctctg-3′	
orexin s	5′-atgaactttccttctacaaag-3′	
orexin as	5′-tcagacatccggaccctccccg-3′	
ox1r s	5′-gtacgagtgggttctcattgc-3′	
ox1r as	5′-gatggcagtcaccagcacatc-3′	
ox2r s	5′-cagtatgtgatgaacactggg-3′	
ox2r as	5′-ctccagcttgatgatctc-3′	
hgprt s	5′-gctgacctgctggattacattaaagcactg-3′	
hgprt as	5′-attcctgaagtactcattatagtcaagggc-3′	
gapdh s	5′-gagctgaacgggaagctcac-3′	
gapdh as	5′-gctacagcaacagggtggtg-3′	
s, sense; as, antisense.		

### Protein analysis by Western blotting

Freshly dissected brains of WT and *fads2*^−/−^ male and female mice were mechanically homogenized in lysate buffer containing protease inhibitor cocktail (Complete; Roche). Protein concentrations were measured using a Pierce BCA protein assay kit (Thermo Scientific).

Protein aliquots (100 μg) were separated by NuPAGE 4–12% Bis-Tris gels and transferred to nitrocellulose membrane using the NuPAGE Western blot system (Invitrogen). Blots were immunostained overnight at 4°C with respective antibodies: anti-fatty acid amide hydrolase (FAAH) (1:200, #ab54615, RRID:AB_2101890) and anti-CB2 from Abcam (1:200, #ab3561, RRID:AB_303908); anti-CB1 (1:200, #sc10066, RRID:AB_637711) and anti-transient receptor potential vallinoid 1 cation channel 1 (TRPV1) from Santa Cruz (1:100, #sc398417, RRID:AB_637711); OrexinR from Alomone Labs (1:200, #AOR-001, RRID:AB_2040048); and anti-α tubulin from Sigma-Aldrich (1:12,000, #T6074, RRID:AB_477582). After washing, HRP-conjugated secondary antibodies were used and detected with the ECL system. Signals were quantified by densitometry using the ImageJ2 program (RRID:SCR_003070).

### Histology and immunohistochemistry

WT and *fads2*^−/−^ male and female mice were perfused from the left ventricle with 25 ml of PBS and with 50 ml of PBS-buffered 4% paraformaldehyde for paraffin embedding and processing for immunofluorescence-microscopy. Endogenous peroxidase activity was blocked with 0.3% H_2_O_2_/methanol for 30 min at room temperature before peroxidase staining of coronal sections (5 μm). Sections were permeabilized with 0.5% Triton X-100/PBS at 4°C, blocked with 3% BSA/0.1% Triton X-100/PBS, and treated with respective antibody dilutions in 3% BSA/0.1% Triton X-100/PBS at 4°C overnight: anti-FAAH from Abcam (1:400, #ab54615, RRID:AB_2101890); anti-CB1 from Santa Cruz (1:200, #sc10066, RRID:AB_637711), and OrexinR from Alomone Labs (1:100, #AOR-001, RRID:AB_2040048).

Sections were washed with PBS/0.05% Tween20, incubated with Cy3-conjugated secondary IgG antibody (Jackson Immuno Research) for 1 h at 37°C, washed with PBS/0.05% Tween20, and immunostained with Affinity purified rabbit polyclonal or monoclonal antibodies and HRP with DAB substrate (Roche #1718096) in the peroxidase reaction. A Zeiss microscope, Axio Imager.M1, and the AxioVision imaging software (RRID:SCR_002677) were used for fluorescence microscopy and a Slide Scanner Leica SCN400 and the Aperio ImageScope software (RRID:SCR_014311) for peroxidase microscopy.

### Thermal nociception test

The hot plate test measuring thermal nociception was carried out following established procedures (24). The plate temperature was maintained at 54–55°C. Cut-off time was set at 60 s. WT and *fads2*^−/−^ female mice were immediately removed from the hot plate when the pain threshold was reached.

### Statistical analysis

Results are expressed as mean ± SEM. Statistical analysis of differences between individual experimental groups was performed using GraphPad QuickCalcs. Sample sizes are indicated in the figure legends. Student’s unpaired two-tailed *t*-test was used. *P*-values of ≤0.05*, ≤0.01**, and ≤0.001*** were considered significant.

## RESULTS

### Dynamics of the PUFA pattern in the phospholipidome of extra-neuronal and neuronal tissues of *fads2*^−/−^ mice

**Fig. 1.**
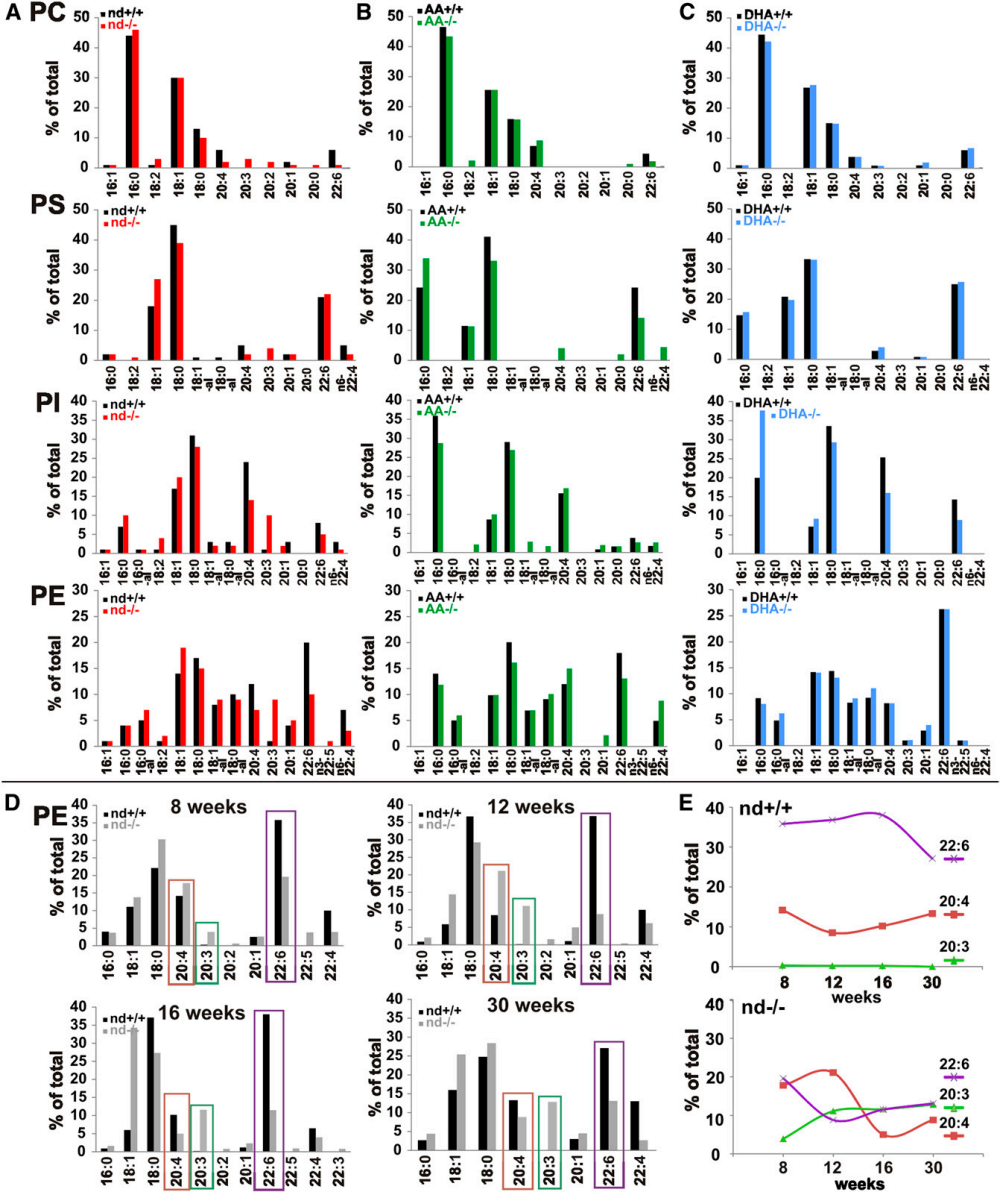
Modification of phospholipidomes of brains of WT and *fads2*^−/−^ mice by ω3- and ω6-PUFA supplemented diet. A–C: Fatty acid pattern of phospholipid classes of brains of WT and *fads2*^−/−^ mice (4 months) on nd (WT, black bars; *fads2*^−/−^, red bars) (A), ω6-AA-diet (WT, black bars; *fads2*^−/−^, green bars) (B), and ω3-DHA-diet (WT, black bars; *fads2*^−/−^, blue bars) (C). D, E: Time-dependent regression of AA (red) and DHA (violet) and increase of ω6-sciadonic acid (green) in PE at 8, 12, 16, and 30 weeks. A pool of n = 3 per genotype was used.

Homozygous male and female *fads2*^−/−^ mice on nd containing the required daily concentration of EFAs are infertile (16). Heterozygous breedings generated cohorts of *fads2*^−/−^ mice. WT and *fads2*^−/−^ mice were kept on sustained nd (nd-WT, nd-*fads2*^−/−^) and either nd supplemented with ω6-AA (ω6-AA-WT, ω6-AA-*fads2*^−/−^) or ω3-DHA (ω3-DHA-WT, ω3-DHA-*fads2*^−/−^) starting after weaning. The PUFA pattern in the lipidome of the brain (**Fig. 1**), liver and serum (supplemental Fig. S1), and of newborn *fads2*^−/−^ progeny reflected that of the *fads2*^+/−^ foster mothers.

GC/MS analysis of the fatty acid pattern of phospholipids revealed regression of ω6-AA and ω3-DHA concentrations in all peripheral tissues of auxotrophic nd-*fads2*^−/−^ siblings within 4 months, here exemplified in total lipid extracts of liver (supplemental Fig. S1B). The liver lipidome of nd-*fads2*^−/−^ mice contained EFAs, but was deprived of ω6- and ω3-LC-PUFAs. Instead, ω6-sciadonic acid was incorporated as surrogate of PUFAs in the diacylglycerol-backbones of all phospholipids. Also, the PUFA pattern of serum phospholipids of nd-, ω6**-**AA-, and ω3**-**DHA-WT and -*fads2*^−/−^ mice (supplemental Fig. S1C–E) showed absence of LC-PUFAs in nd-*fads2*^−/−^ mice (supplemental Fig. S1C).

We next focused on the PUFA pattern of the brain phospholipidomes of adult (4 months) nd-, ω6-AA-, and ω3-DHA-WT and -*fads2*^−/−^ mice, as critical sources of PUFA substrates for on-demand endocannabinoid synthesis. Phospholipid classes were separated by HPTLC, and their FAME substituents identified and quantitated by GC/MS (Fig. 1A–C).

Unlike the systemic depletion of PUFAs in peripheral tissues (supplemental Fig. S1A), PUFA substituents of phospholipid classes in the brains of nd-*fads2*^−/−^ mice decreased to low levels, which persisted during their lifespan (Fig. 1A). ω6-AA- and ω3-DHA-supplemented diets fully replenished the ω6-AA- and ω3-DHA-LC-PUFA concentration of *fads2*^−/−^ brain within 4 months and completely replaced ω6-sciadonic acid in all phospholipid classes (Fig. 1B, C).

Following this surprising observation, we measured the kinetics of the retarded PUFA exchange in the brains of 8-, 12-, 16-, and 30-week-old nd-WT and nd-*fads2*^−/−^ mice, taking PE species as paradigm. ω6-Sciadonic acid increased to a final concentration of 10% of total fatty acids within 12 weeks, ω6-AA and ω3-DHA inversely decreased to 10% after 30 weeks and persisted at this level during the lifespan (Fig. 1D, E).

### Absence of Δ8-desaturase activity in *fads2*-expressing cells prohibits the rescue of AA synthesis

**Fig. 2.**
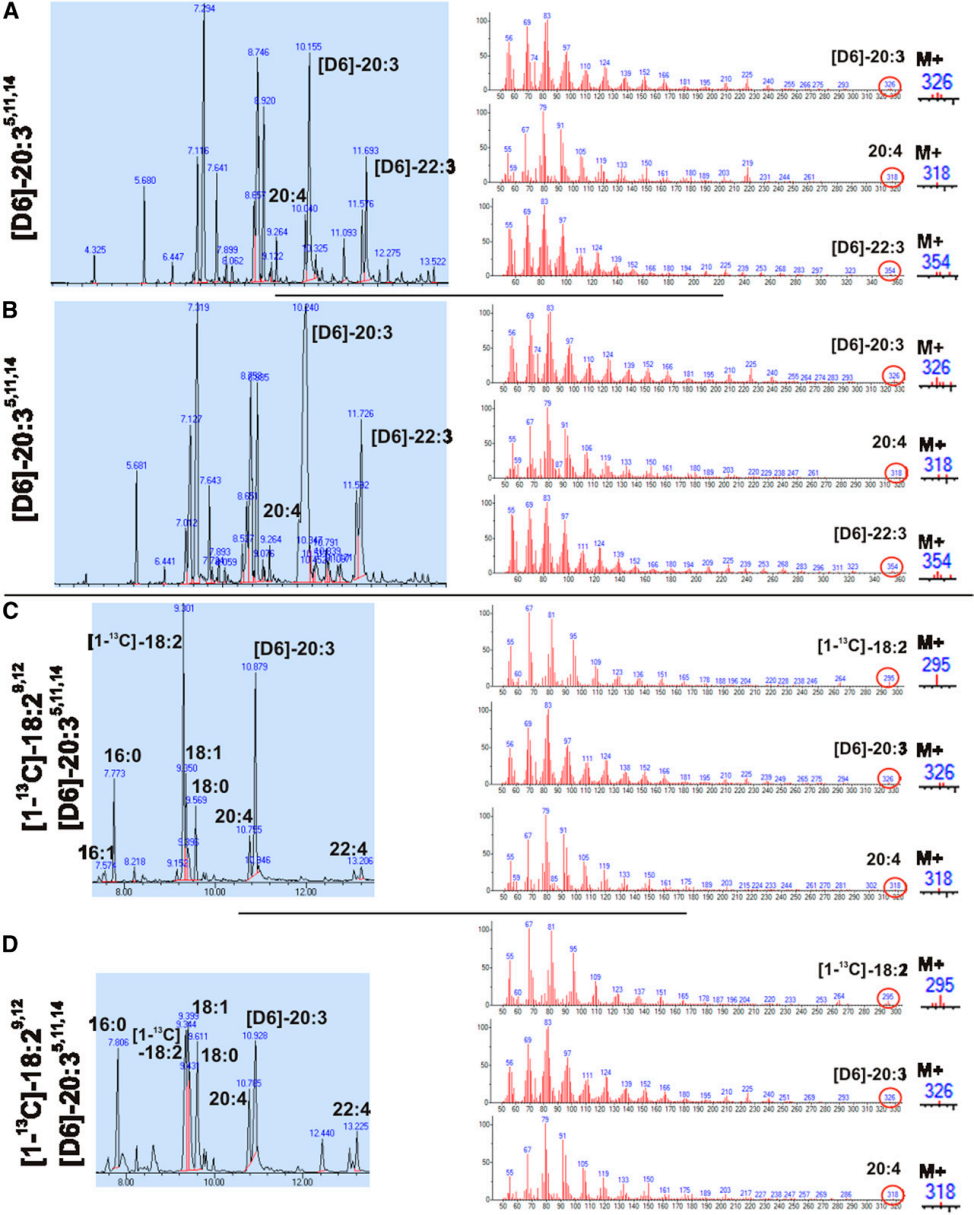
Position specificity of FADS2 activity and absence of Δ8-desaturase activity. A, B: GC-MS of FAMEs of total lipids of mock-transfected (A) and *fads2*-transfected (B) HEK293 cells incubated with [D6]ω6-sciadonic acid. C, D: WT (C) and *fads2*^−/−^ (D) MEFs incubated with [1-^13^C]ω6-linoleic acid and [D6]ω6-sciadonic acid.

We investigated the presence of a Δ8-desaturase in mock- and *fads2*-transfected HEK293 cells, following the uptake, transformation, and utilization of [D6]ω6-sciadonic acid (M^+^326) as substrate in phospholipid biosynthesis. GC/MS analysis documented the absence of [D6]ω6-AA (M^+^324) (**Fig. 2A**, B).

Furthermore, we investigated potential Δ8-desaturase activity associated with FADS2 in WT-MEFs (Fig. 2C) and *fads2*^−/−^-MEFs (Fig. 2D) fed with [1-^13^C]ω6-linoleic acid (M^+^295) and [D6]ω6-sciadonic acid (M^+^326). GC/MS of FAMEs isolated from total lipid extracts showed labeled substrates and [D6]ω6-22:3^7,13,16^ (M^+^354), the elongation product of [D6] ω6-sciadonic acid, but no labeled AA (M^+^318). These results preclude Δ8-desaturase activity either as enzyme entity or associated with FADS2, underlining the position specificity of Δ6-desaturase (FADS2).

### Two novel endocannabinoids in PUFA synthesis deficiency: *N*-eicosa-5,11,14-trienoyl-ethanolamide and 2-eicosa-5,11,14-trienoyl-glycerol

**Fig. 3.**
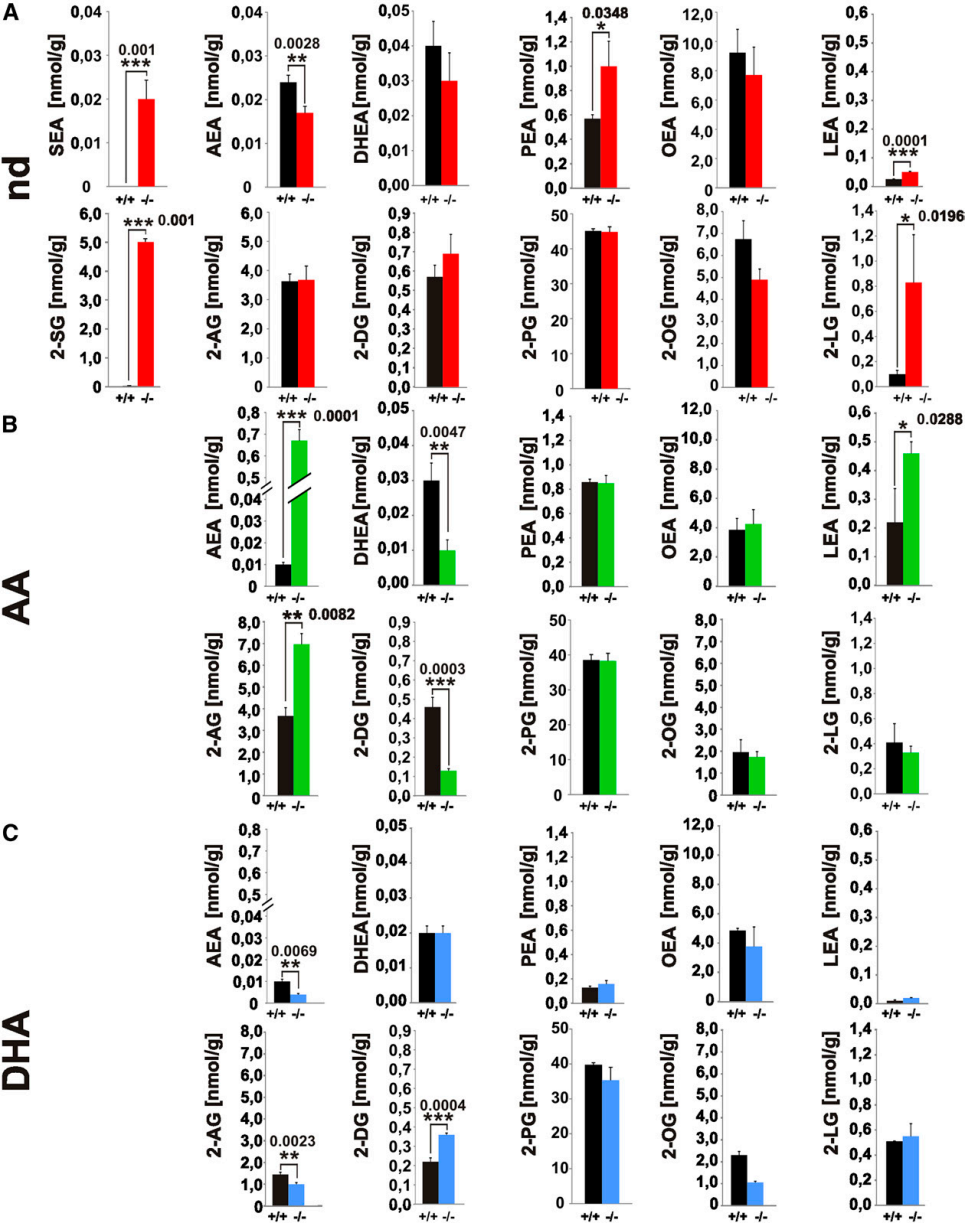
Novel endocannabinoids 20:3^5,11,14^-(sciadonoyl)-ethanolamide (SEA) and 2-20:3^5,11,14^-(sciadonoyl)-glycerol (2-SG) in the endocannabinoid pool of brains of nd-*fads2*^−/−^ mice. A–C: Separation and quantification of endocannabinoids in lipid extracts of brain of nd- (WT, black bars; *fads2*^−/−^, red bars) (A), ω6-AA- (WT, black bars; *fads2*^−/−^, green bars) (B), and ω3-DHA- (WT, black bars; *fads2*^−/−^, blue bars) (C) -WT and -*fads2*^−/−^ mice by LC-MS/MS. Data represent mean ± SEM. Two-tailed Student’s *t*-test; *P ≤ 0.05, **P ≤ 0.01, ***P ≤ 0.001 were considered significant; n = 8 per genotype. Sciadonoyl-ethanolamide (SEA), 2-sciadonoyl-glycerol (2-SG), 2-oleoyl-glycerol (2-OG), 2-linoyl-glycerol (2-LG), 2-arachidonoyl-glycerol (2-AG).

The pool of NAEs and 2-MAGs in lipid extracts of brain and serum of adult nd-, ω6-AA-, and ω3-DHA-WT and -*fads2*^−/−^ mice was analyzed by LC-MS/MS (**Fig. 3**, supplemental Fig. S2). We discovered two novel endocannabinoids, *N*-eicosa-5,11,14-trienoyl-ethanolamide (SEA) and 2-eicosa-5,11,14-trienoyl-glycerol (2-SG), derived from the uncommon ω6-sciadonic acid in brain and serum of nd*-fads2*^−/−^ mice. SEA and 2-SG were present in nearly stoichiometric concentrations to AEA and 2-AG in nd-WT brain and serum.

The localization and double bond structure of the precursor ω6-sciadonic acid was confirmed by GC-MS analysis of the 4,4-dimethyloxazoline (DMOX) derivatives, the fragment *m/z* 154 indicating the Δ5 *cis* double bond position (25), which proved identical to the [1-^13^C]- and [D6]-labeled ω6-sciadonic acid obtained by chemical synthesis (supplemental Fig. S3I, J). LC-MS/MS extracted ion chromatography of the lipid extract from brains of nd-*fads2*^−/−^ mice detected SEA, transition 350.3–62.06 Da (supplemental Fig. S3A), and its TOF product 350.3 (supplemental Fig. S3B), and 2-SG, transition 381.3–289.3 Da (supplemental Fig. S3C) and TOF product 381.3 (supplemental Fig. S3D).

LC-MS/MS analysis of endocannabinoids in the brain lipidome of ω6-AA-*fads2*^−/−^ mice revealed 30-fold elevated AEA and 2-fold 2-AG concentrations (Fig. 3C), but low levels of docosahexaenoyl-ethanolamide (DHEA) and 2-docosahexaenoyl-glycerol (2-DG) compared with nd-*fads2*^−/−^ brain. Of note is the 10-fold higher concentration of LEA in ω6-AA-WT and -*fads2*^−/−^ mice. Endocannabinoids in the brain lipidomes of ω3-DHA-WT and -*fads2*^−/−^ mice revealed significantly lower AEA and 2-AG concentrations compared with the two nd cohorts.

The pattern of serum endocannabinoids reflects that of brain, although their concentration is 10- to 100-fold lower (supplemental Fig. S2).

### PUFAs in the regulation of gene and protein expression of the endocannabinoid and orexinergic systems

**Fig. 4.**
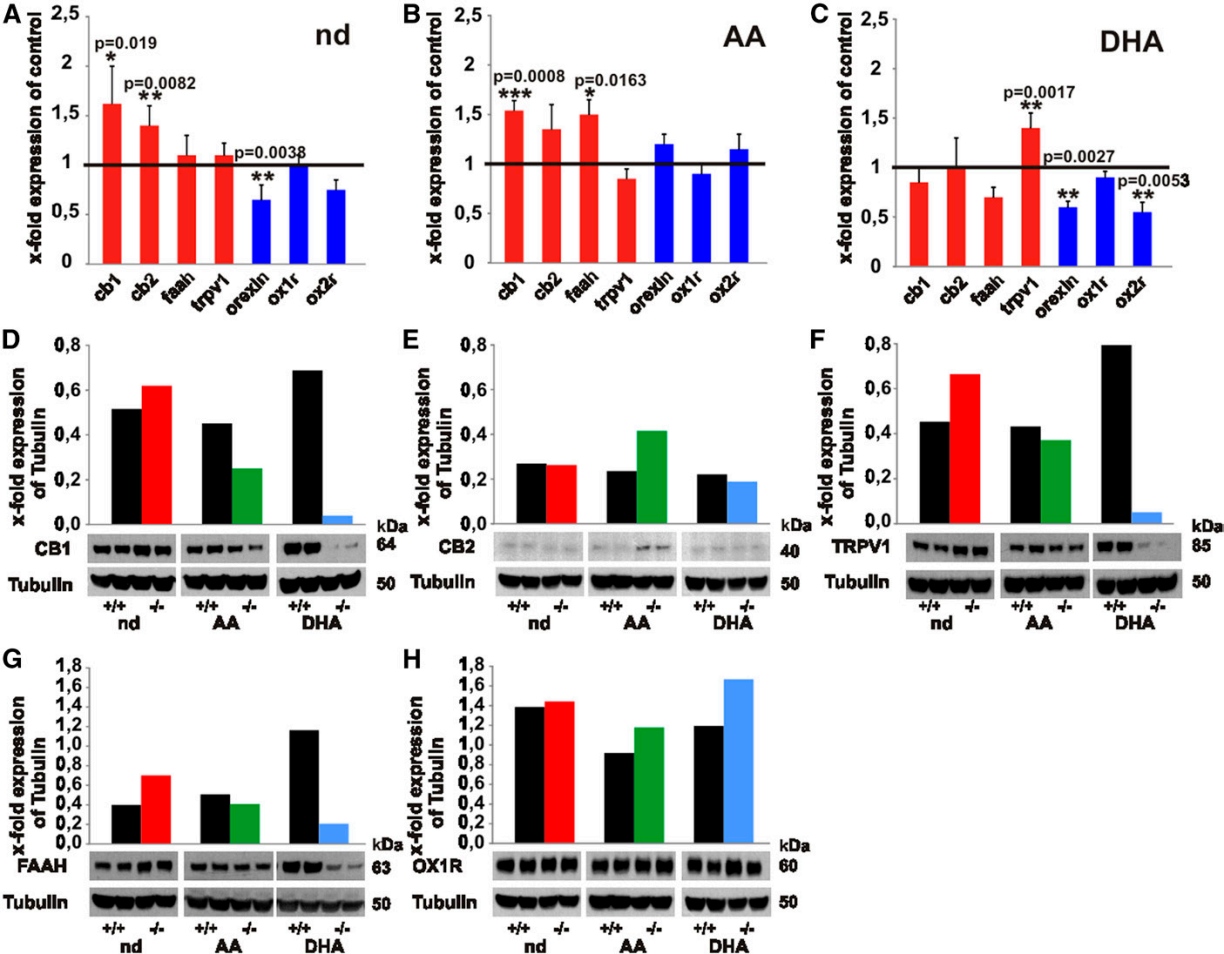
Transcription of genes and translation of proteins operative in the endocannabinoid and orexin systems in brain of nd-, ω6-AA-, and ω3-DHA-WT and -*fads2*^−/−^ mice. A–C: Real-time PCR of cannabinoid receptor 1 (cb1) and 2 (cb2), orexin, and orexin receptor 1 (ox1r) and ox2r of cRNA of brain of nd-WT and -*fads2*^−/−^ mice (A), ω6-AA-WT and -*fads2*^−/−^ mice (B), and ω3-DHA-WT and -*fads2*^−/−^ mice (C) (n = 5 per genotype). Data represent mean ± SEM. *P ≤ 0.05, **P ≤ 0.01, ***P ≤ 0.001 were considered significant. D–H: Western blot analysis of protein lysates of brain nd- (WT, black bars; *fads2*^−/−^, red bars), ω6-AA- (WT, black bars; *fads2*^−/−^, green bars), and ω3-DHA- (WT, black bars; *fads2*^−/−^, blue bars) -WT and -*fads2*^−/−^ mice using anti-CB1- (D), anti-CB2- (E), anti-TRPV1- (F), anti-FAAH- (G), and anti-OrexinR1- (H) antibody (n = 3).

Quantitative real-time PCR of stationary RNA levels indicated significantly elevated expression of *cb1* and *cb2* in nd*-fads2*^−/−^ mice (**Fig. 4A**) and of *cb1 *in ω6-AA*-fads2*^−/−^ mice (Fig. 4B). Expression of *cb1* remained unchanged in ω3-DHA-*fads2*^−/−^ mice (Fig. 4C), but there was a marked upregulation of the expression of the *trpv1* gene. *Orexin* and *orexin receptor 2* (*ox2r*) expressions were suppressed in ω3-DHA-*fads2*^−/−^ brain (Fig. 4C).

Western blot analysis of proteins in brain lysates of nd-*fads2*^−/−^ mice revealed upregulation of the NAE degrading enzyme, FAAH, and of TRPV1 receptor (Fig. 4F, G). In ω6-AA-*fads2*^−/−^ mouse brain, CB1 was downregulated (Fig. 4D*)* and, inversely, CB2 was upregulated (Fig. 4E). Brains of ω3-DHA-*fads2*^−/−^ mice synthesized low concentrations of CB1, TRPV1, and FAAH (Fig. 4D, F, G*)*.

### Regional distribution of CB1 and OX1R expression in *fads2*^−/−^ mice

**Fig. 5.**
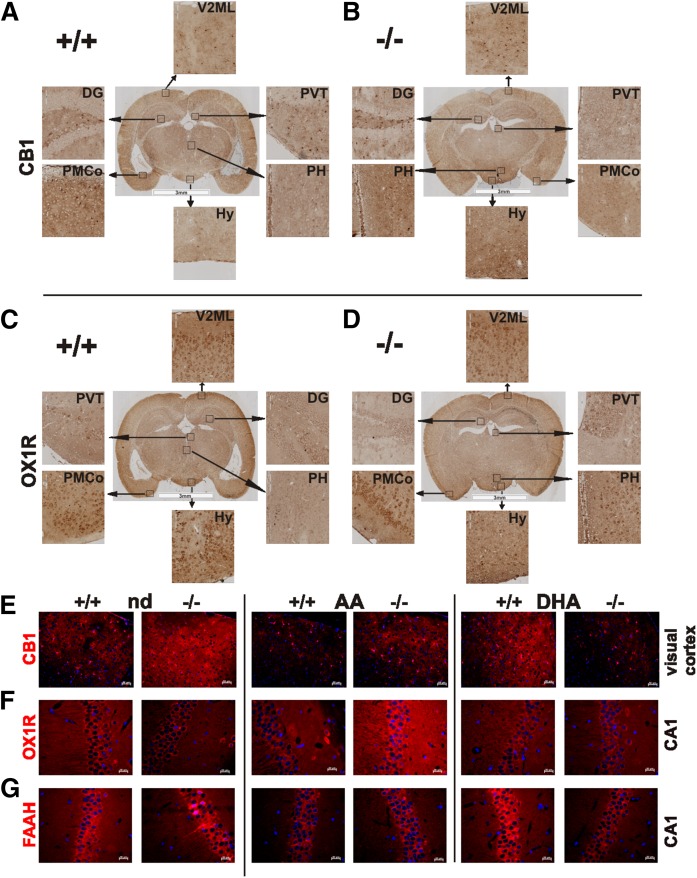
Distribution of CB1 and OX1R in coronal section of brain of nd-WT (A, C) (Bregma −2.8 mm) and -*fads2*^−/−^ (B, D) (Bregma −2.3 mm) mice. A–D: Sections were stained with anti-CB1- (A, B) and anti-OX1R-antibodies (C, D) and HRP-tagged secondary antibody (n = 3). Encased areas are magnified regions of dentate gyrus (DG), hypothalamus (Hy), posterior hypothalamus (PH), posteromedial cortical amygdaloid nucleus (PMCo), paraventricular nucleus of the thalamus (PVT), subparaventricular zone (SBPV), and visual cortex 2 mediolateral (V2ML). E–G: IHC localization of CB1, OXR1, and FAAH in sections of brain of nd-, ω6-AA-, and ω3-DHA-WT and *-fads2^−/−^* mice. Visual cortex with anti-CB1 (E), CA1 with anti-OX1R (F), and CA1 with anti-FAAH (G). Cy3-labeled secondary antibody was used (n = 3).

Regional expression of CB1 and OX1R, representing the ECS and orexinergic system of brains of nd-WT and -*fads2*^−/−^ mice, was visualized in coronal sections by IHC, using anti-CB1 and anti-OX1R antibodies and anti-HRP-labeled secondary antibody (**Fig. 5A**–D). Neurons in hypothalamus, visual cortex 2 mediolateral, and dentate gyrus showed pronounced CB1 (Fig. 5A, B) and OX1R expression in posterior hypothalamus of nd-*fads2*^−/−^ mice compared with nd-WT mice (Fig. 5C, D).

IHC signal intensities in images of fluorescence-stained coronal sections recorded under identical parameters indicated enhanced expression of CB1 in nd-*fads2*^−/−^ visual cortex, FAAH in the nd-*fads2*^−/−^ CA1 region, and OX1R in the CA1 region of ω6-AA-*fads2*^−/−^ brain. Fluorescence intensities of CB1 in the visual cortex and of CA3 and FAAH in the CA1 and CA3 regions in ω3-DHA-WT brain were strikingly higher than in ω3-DHA-*fads2*^−/−^ brain (Fig. 5E–G).

### Activator function of SEA and 2-SG in *cb1*-expressing HEK293 cells

Mock- and *cb1*-transfected HEK293 cells were characterized as described (supplemental Fig. S4). Semi-confluent cultures were exposed to SEA and 2-SG and compared with AEA and 2-AG. Their function in activating the G_i/o_ protein-coupled CB1 was tested by recording changes in intensities of intracellular [Ca^2+^] transient-induced Fluo-4 fluorescence.

**Fig. 6.**
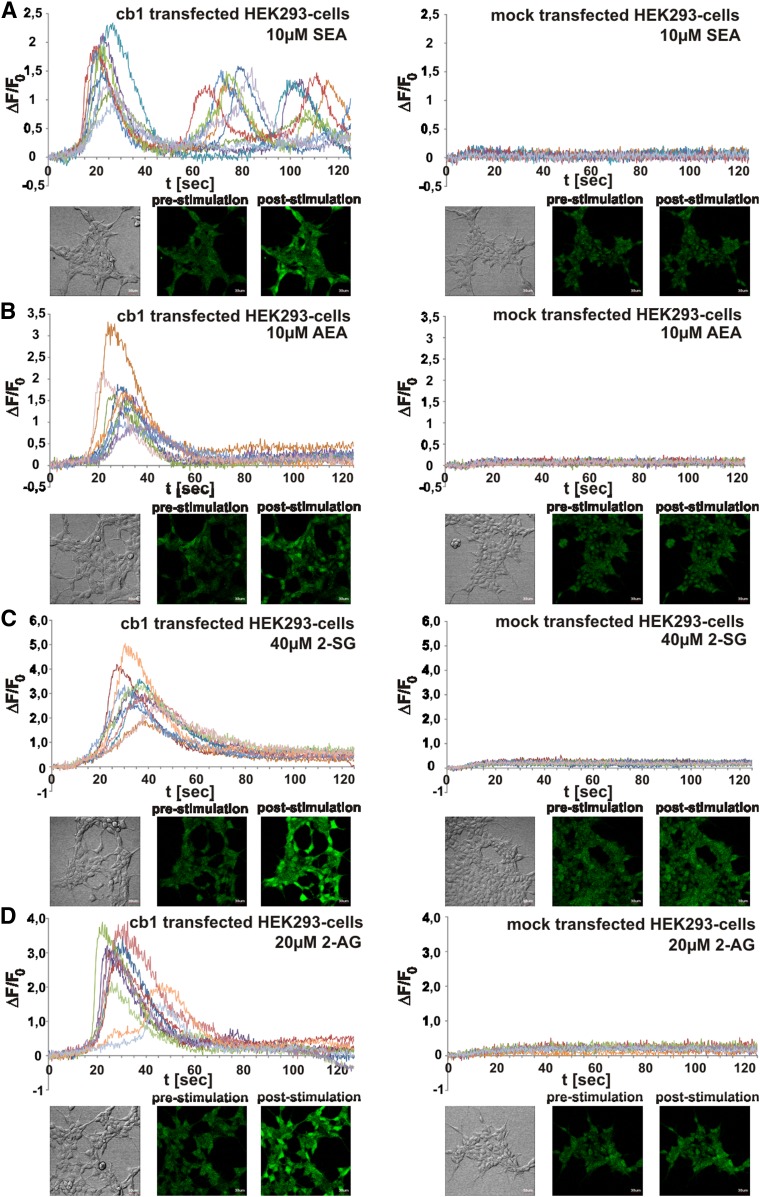
Analysis of activator function of endocannabinoids SEA and 2-SG compared with AEA and 2-AG in *cb1*- and mock-transfected HEK293 cells by comparative Fluo-4 imaging. A–D: Fluorescent signals of CB1 agonist-induced Ca^2+^ transients in each of 10 randomly selected HEK293 cells were monitored after SEA (A), AEA (B), 2-SG (C), and 2-AG (D) addition. Time-dependent corrected relative intensities (ΔF/F_0_) and pictures at zero time (pre-stimulation) and at peak activity (post-stimulation) are presented (n = 10).

Comparable time-dependent corrected fluorescent intensities (ΔF/F_0_) induced by AEA and 2-AG and the novel SEA and 2-SG ligands were recorded in single *cb1*-transfected HEK293 cells (**Fig. 6A**–D).

### Cannabimimetic behavior effects in nd-, ω6-AA-, and ω3-DHA-WT and -*fads2*^−/−^ mice

We complemented the quantitative biochemical analysis of the different endocannabinoid patterns of nd-, ω6-AA-, and ω3-DHA-WT and -*fads2*^−/−^ mice by canonical cannabimimetic tests.

**Fig. 7.**
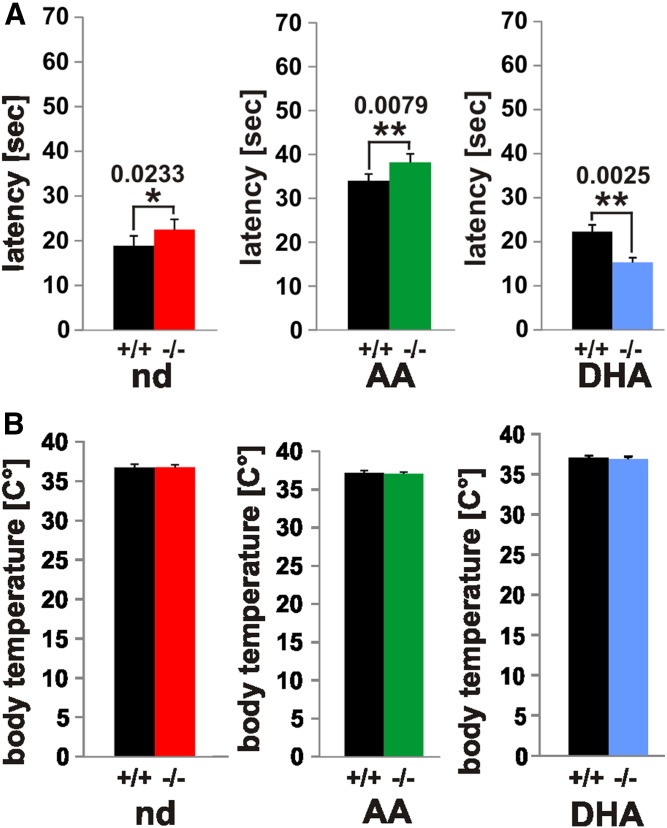
Nociception and body temperature of nd-, ω6-AA-, and ω3-DHA-WT and -*fads2*^−/−^ mice. A, B: Hot plate test [latency (s)] (A) and rectal temperature (°C) (B) of nd- (WT, black bars; *fads2*^−/−^, red bars), ω6-AA- (WT, black bars; *fads2*^−/−^, green bars), and ω3-DHA- (WT, black bars; *fads2^−/−^*, blue bars) -WT and -*fads2*^−/−^ mice. Data represent mean ± SEM. *P ≤ 0.05, **P ≤ 0.01, ***P ≤ 0.001 were considered significant (n = 8).

We tested nociception in the three mouse cohorts in the hot plate test. Prolonged latency was observed in nd*-fads2*^−/−^ and ω6-AA*-fads2*^−/−^ mice compared with their WT controls (**Fig. 7A**). Thermo-sensitivity of ω3-DHA*-fads2*^−/−^ mice was significantly higher compared with ω3-DHA-WT mice. The most notable observation was prolonged latency in ω6-AA-WT and AA-*fads2*^−/−^ mice to almost twice that of nd-WT and nd-*fads2*^−/−^ and ω3-DHA-WT and DHA-*fads2*^−/−^ mice (Fig. 7A).

Rectal temperatures of WT mice and *fads2*^−/−^ mice in the three cohorts remained unchanged (Fig. 7B).

## DISCUSSION

The comparative analysis of the turnover of PUFAs in the phospholipidomes of CNS and extraneuronal tissues of WT and *fads2*^−/−^ mice revealed the postnatal systemic complete depletion of LC-PUFAs in peripheral tissues, unlike the loss of LC-PUFA substituents and inverse substitution by ω6-sciadonic acid in the diacylglycerol backbone of the phospholipidome of brain, which commenced after weaning and proceeded to a low but constant level and persisted during the lifespan.

The systemic absence of ω6-AA in the adult *fads2*^−/−^ mouse precludes the desaturation of ω6-sciadonic acid to ω6-AA by a Δ8-desaturase as enzyme entity. Tracing experiments with *fads2*^−/−^ MEFs, using labeled ω6-linoleic acid and ω6-sciadonic acid as substrates, detected no synthesis of labeled ω6-AA. Absence of the transformation of ω6-sciadonic acid to ω6-AA in *fads2*-overexpressing HEK293 cells excluded Δ8-desaturase activity. This underlines the position specificity of the enzyme of FADS2.

Sustained stringent PUFA-supplemented diets of three cohorts led to a homeostatic PUFA pattern of phospholipids of CNS membrane bilayers, which served as substrate donor for endocannabinoid synthesis. We discovered two novel endocannabinoids from brains of nd-*fads2*^−/−^ mice, which were characterized as *N*-eicosa-5Z,11Z,14Z-trienoyl-(sciadonoyl)-ethanolamide (SEA) and 2-eicosa-5Z,11Z,14Z-trienoyl-(sciadonoyl)-glycerol (2-SG) derived from precursor ω6-sciadonic acid. They are equivalent surrogates to their physiological ω6-AA-derived endocannabinoids, AEA and 2-AG, in vivo and as ligands of CB1 in *cb1*-overexpressing HEK293 cells in culture.

This suggests dietary ω6-linoleic acid supply to be sufficient, maintaining homeostasis of the mammalian ECS.

Gene and protein expression of key players in the ECS and the orexinergic system in brains of nd-, AA-, and DHA*-fads2*^−/−^ cohorts indicated remarkable changes in cannabimimetic effects of the novel endocannabinoids and their function in their connectivity to the orexinergic system.

The synthesis of endocannabinoid ligands of CB1 in the ECS of the CNS in the *fads2*^−/−^ mouse depends on the dietary supply of EFAs and preformed LC-PUFAs. The PUFA ratio in current Western diet is regarded as a critical nutritional parameter for numerous cardiovascular, metabolic, and neurodegenerative diseases and is regarded as a putative epigenetic factor (1, 3, 4).

This study elaborates the impact of the supply of ω3- and ω6-LC-PUFAs as crucial precursors in endocannabinoid synthesis using the auxotrophic Δ6-desaturase-deficient (*fads2*^−/−^) mouse in unbiased feeding experiments. PUFA-deficient *fads2*^−/−^ mice synthesize ω6-sciadonic acid in an unusual pathway, utilizing linoleic acid for chain elongation followed by Δ5-desaturation. ω6-AA and ω3-DHA feeding overcomes ω6-sciadonic acid synthesis systemically (18).

### Incomplete deprivation of ω3- and ω6-LC-PUFAs from the *fads2*^−/−^ CNS phospholipidome

Kinetic studies revealed loss of the ω3- and ω6-LC-PUFA substituents in the phospholipidome of extra-neuronal tissues (supplemental Fig. S1) and complete substitution by ω6-sciadonic acid.

Unexpectedly, the phospholipidome of brains of adult nd-*fads2*^−/−^ mice contained persisting ω3- and ω6-PUFA substituents (Fig. 1A). Phospholipid class-specific LC-PUFA depletion followed a linear regression starting after weaning and reached low but constant levels within 6–7 months, which persist during the lifespan (Fig. 1D,E).

The kinetics clearly indicated the caveats of short-time feeding experiments, aiming at manipulating the lipid bilayer architecture of neuronal membrane systems as microenvironment of functionally diverse integral membrane proteins.

### Absence of Δ8-desaturation of ω6-sciadonic acid to ω6-AA in *fads2*^−/−^ mice

Δ8-Desaturase activity has been attributed to Δ6-desaturase (FADS2) in an alternative pathway leading to ω6-AA (26). This study, however, is at variance with this observation. *Fads2*-overexpressing HEK293 cells are unable to desaturate [D6]ω6-sciadonic acid to [D6]ω6-AA, indicating absolute position-specific Δ6-desaturase activity. The *fads2*^−/−^ mouse also lacks Δ8-desaturase activity as an enzyme entity, which is supported by GC/MS analysis of FAMEs of total lipid extracts of *fads2*^−/−^ MEFs, unable to desaturate [D6]ω6-sciadonic acid to [D6]ω6-AA (Fig. 2).

### Structure-function relationship of SEA and 2-SG as CB1 ligands

A remarkable neuronal response to LC-PUFA deficiency was the synthesis of two novel endocannabinoids, which were identified as SEA and 2-SG and quantified by LC/MS/MS (Fig. 3; supplemental Figs. S2, S3). They share identical structures of their carboxyl ends, including the Δ5 *cis*-double bond position with most active endocannabinoids’ AEA and 2-AG.

Another abnormal eicosatrienoic acid, ω9-20:3^5,8,11^ (“Mead acid”), derived from the monoenoic oleic acid (27), occurs in EFA deficiency. Endocannabinoids derived from this eicosatrienoic acid, ω9-20:3^5,8,11^-ethanolamide, and 2-20:3^5,8,11^-glycerol have been described as highly active endocannabinoids (28, 29). These studies also emphasize the Δ5-double bound position as a lead structure, critical for endocannabinoid activity (15).

In view of the discovery of SEA and 2-SG in the CNS of the *fads2*^−/−^ mouse and their stoichiometric surrogate function of AEA and 2-AG, we further proved their ligand properties and activator function of the G protein-coupled CB1 receptor of the ECS in *cb1*-transfected HEK293 cells by monitoring the Fluo-4 fluorescence intensities evoked by Ca^2+^ release. Ligand activity of SEA and 2-SG was comparable with that of AEA and 2-AG (Fig. 6).

Our findings expand the range of CB1 orthosteric ligand structures and underline their important molecular conformation essential for the binding and activation of the receptor. Our study provides valuable in vivo and in vitro systems for the translation of endocannabinoid structures designed and optimized in in silico analysis at the atomic level of modulators docking to crystallographic structures of the CB1 receptor (30–32).

### Modification of the endocannabinoid pattern of brains of WT and *fads2*^−/−^ mice by sustained feeding experiments

The surprisingly stable residual LC-PUFA pool in the phospholipidome led to the synthesis of the LC-PUFA related endocannabinoids in nd-*fads2*^−/−^ brain (Fig. 3A). Sustained dietary supply of ω6-AA suppresses the utilization of ω3-PUFA in *fads2*^−/−^ mice and consequently led to reduced DHEA and 2-DG levels in phospholipids, in agreement with previous studies (33). Supplementation of ω3-DHA in *fads2*^−/−^ mice suppressed the utilization of ω6-PUFA and therefore reduces AEA and 2-AG levels. Reduction of AEA and 2-AG concentrations has also been observed in short-term dietary supplementation of ω3-DHA and its impact on the ECS in brain and plasma in WT-CD1 mice reported previously (34).

### Connectivity of the ECS and orexinergic system in *fads2*^−/−^ mice

The nd-*fads2*^−/−^ mice (4 months) have a lean phenotype; ω6-AA- and ω3-DHA-*fads2*^−/−^ mice develop pronounced obesity (18). The observed low levels of 2-AG in ω3-DHA-WT and -*fads2*^−/−^ mice might contribute to the modulation of the orexinergic system, inducing enhanced lipogenesis in the DHA-*fads2*^−/−^ mice. HRP- and IHC-stained coronal sections of nd-WT and -*fads2*^−/−^ brains display overlapping expression of CB1 and OX1R neurons (Fig. 5).

It is tempting to speculate that reduced availability of AEA and 2-AG in DHA-*fads2*^−/−^ mice suppresses CB1, TRPV1, and FAAH synthesis, documented in WB and IHC (Figs. 4, 5).

In the brains of nd-*fads2*^−/−^ mice, the two novel endocannabinoids took over the function of AEA and 2-AG in the retrograde action as ligands of presynaptic CB1 on orexinergic neurons in different areas of brain (lateral hypothalamus, hippocampus, arcuate nucleus. The release of inhibitory GABAergic inputs promotes increased appetite and feeding and increased body fat mass, resulting in obesity, and excitatory glutamatergic inputs, suppressing hunger and induce hypophagy (14, 35, 36).

### Cannabimimetic effects of novel endocannabinoids in FADS2 deficiency

PEA, like AEA and 2-AG, is known to attenuate nociception (37, 38). The decrease of PEA, AEA, and 2-AG in the brains of ω3-DHA-WT and ω3-DHA-*fads2*^−/−^ mice explains the pro-nociceptive effect in *fads2*^−/−^ mice but not in WT mice. ω3-DHA supplementation suppresses the synthesis of ω6-AA to lower levels of AA, the precursor substrate of AEA and 2-AG. Reduction of the CB1 agonists, AEA and 2-AG, causes enhanced thermosensitivity (34, 39–44). The reduced availability of ω6-AA as substrate of the cyclooxygenase and lipoxygenase pathways impairs the synthesis of inflammatory eicosanoids (45). The mutual regulation of ω3- and ω6-LC-PUFA synthesis by diet supplied or from the endogenous synthesis from EFAs on the level on desaturases, elongases, and acyl-transferases has been extensively studied previously (46–48).

Increased LEA inhibits the activity of FAAH (10-fold), which prolongs AEA activity in ω6-AA-WT and -*fads2*^−/−^ mice (49, 50). Anti-nociception of ω6-AA-WT and -*fads2*^−/−^ mice is almost twice that of nd- and ω3-DHA-WT and *-fads2*^−/−^ mice. Body temperature of WT and *fads2*^−/−^ mice remained unchanged.

Our study delineates the individual role of EFAs and ω3- and ω6-LC-PUFAs as essential dietary precursors in the synthesis of endocannabinoid ligands of CB1/2 receptors in the ECS, cooperating with the orexinergic neuronal network. However, beyond the regulation of the feeding behavior for maintaining energy homeostasis by the ECS and orexinergic system, additional relevant metabolic targets of PUFAs or their derivatives leading to the lean (anti-obese) phenotype of the LC-PUFA-deficient nd-*fads2*^−/−^ mice and the obese phenotype of the ω3-DHA-*fads2*^−/−^ mice have to be addressed by future investigations. PUFAs as well as derived eicosanoids and endocannabinoids have been identified as CB1 and CB2 independent, directly binding ligands of subtypes of the nuclear receptor superfamily. ω3-DHA and ω6-AA have been recognized as ligands of nuclear receptor RxR in brain (51, 52) and PPARα, -β, and -γ (53, 54).

This study predisposes the *fads2*^−/−^ mouse mutant as an unbiased mouse model for the discovery of therapeutically useful CB1 agonists and antagonists in the therapy of CNS-dysregulated feeding behavior and for metabolic diseases, including obesity and diabetes.

## Supplementary Material

Supplemental Data

## References

[1] Broadhurst C. L., Wang Y., Crawford M. A., Cunnane S. C., Parkington J. E., and Schmidt W. F. 2002 Brain-specific lipids from marine, lacustrine, or terrestrial food resources: potential impact on early African Homo sapiens. Comp. Biochem. Physiol. B Biochem. Mol. Biol. 131 653–673. 11923081 10.1016/s1096-4959(02)00002-7

[2] Simopoulos A. P. 2008 The importance of the omega-6/omega-3 fatty acid ratio in cardiovascular disease and other chronic diseases. Exp. Biol. Med. (Maywood). 233: 674–688. 18408140 10.3181/0711-MR-311

[3] Breslow J. L. 2006 n-3 fatty acids and cardiovascular disease. Am. J. Clin. Nutr. 83(6, Suppl) 1477S–1482S. 16841857 10.1093/ajcn/83.6.1477S

[4] Crawford M. A., and Broadhurst C. L. 2012 The role of docosahexaenoic and the marine food web as determinants of evolution and hominid brain development: the challenge for human sustainability. Nutr. Health. 21: 17–39. 22544773 10.1177/0260106012437550

[5] Wiktorowska-Owczarek A., Berezinska M., and Nowak J. Z. 2015 PUFAs: structures, metabolism and functions. Adv. Clin. Exp. Med. 24: 931–941. 26771963 10.17219/acem/31243

[6] Marventano S., Kolacz P., Castellano S., Galvano F., Buscemi S., Mistretta A., and Grosso G. 2015 A review of recent evidence in human studies of n-3 and n-6 PUFA intake on cardiovascular disease, cancer, and depressive disorders: does the ratio really matter? Int. J. Food Sci. Nutr. 66: 611–622. 26307560 10.3109/09637486.2015.1077790

[7] Matsuda L. A., Lolait S. J., Brownstein M. J., Young A. C., and Bonner T. I. 1990 Structure of a cannabinoid receptor and functional expression of the cloned cDNA. Nature. 346: 561–564. 2165569 10.1038/346561a0

[8] Devane W. A., Hanus L., Breuer A., Pertwee R. G., Stevenson L. A., Griffin G., Gibson D., Mandelbaum A., Etinger A., and Mechoulam R. 1992 Isolation and structure of a brain constituent that binds to the cannabinoid receptor. Science. 258: 1946–1949. 1470919 10.1126/science.1470919

[9] Mechoulam R., Ben-Shabat S., Hanus L., Ligumsky M., Kaminski N. E., Schatz A. R., Gopher A., Almog S., Martin B. R., Compton D. R., 1995 Identification of an endogenous 2-monoglyceride, present in canine gut, that binds to cannabinoid receptors. Biochem. Pharmacol. 50: 83–90. 7605349 10.1016/0006-2952(95)00109-d

[10] Chávez A. E., Chiu C. Q., and Castillo P. E. 2010 TRPV1 activation by endogenous anandamide triggers postsynaptic long-term depression in dentate gyrus. Nat. Neurosci. 13: 1511–1518. 21076423 10.1038/nn.2684 PMC3058928

[11] Di Marzo V., Fontana A., Cadas H., Schinelli S., Cimino G., Schwartz J. C., and Piomelli D. 1994 Formation and inactivation of endogenous cannabinoid anandamide in central neurons. Nature. 372: 686–691. 7990962 10.1038/372686a0

[12] Sugiura T., Kondo S., Sukagawa A., Tonegawa T., Nakane S., Yamashita A., and Waku K. 1996 Enzymatic synthesis of anandamide, an endogenous cannabinoid receptor ligand, through N-acylphosphatidylethanolamine pathway in testis: involvement of Ca(2+)-dependent transacylase and phosphodiesterase activities. Biochem. Biophys. Res. Commun. 218: 113–117. 8573114 10.1006/bbrc.1996.0020

[13] Dinh T. P., Carpenter D., Leslie F. M., Freund T. F., Katona I., Sensi S. L., Kathuria S., and Piomelli D. 2002 Brain monoglyceride lipase participating in endocannabinoid inactivation. Proc. Natl. Acad. Sci. USA. 99: 10819–10824. 12136125 10.1073/pnas.152334899 PMC125056

[14] Koch M. 2017 Cannabinoid receptor signaling in central regulation of feeding behavior: a mini-review. Front. Neurosci. 11: 293. 28596721 10.3389/fnins.2017.00293 PMC5442223

[15] Reggio P. H. 2010 Endocannabinoid binding to the cannabinoid receptors: what is known and what remains unknown. Curr. Med. Chem. 17: 1468–1486. 20166921 10.2174/092986710790980005 PMC4120766

[16] Stoffel W., Holz B., Jenke B., Binczek E., Gunter R. H., Kiss C., Karakesisoglou I., Thevis M., Weber A. A., Arnhold S., 2008 Delta6-desaturase (FADS2) deficiency unveils the role of omega3- and omega6-polyunsaturated fatty acids. EMBO J. 27: 2281–2292. 19172737 10.1038/emboj.2008.156 PMC2529369

[17] Stroud C. K., Nara T. Y., Roqueta-Rivera M., Radlowski E. C., Lawrence P., Zhang Y., Cho B. H., Segre M., Hess R. A., Brenna J. T., 2009 Disruption of FADS2 gene in mice impairs male reproduction and causes dermal and intestinal ulceration. J. Lipid Res. 50: 1870–1880. 19351970 10.1194/jlr.M900039-JLR200 PMC2724775

[18] Stoffel W., Hammels I., Jenke B., Binczek E., Schmidt-Soltau I., Brodesser S., Odenthal M., and Thevis M. 2014 Obesity resistance and deregulation of lipogenesis in Delta6-fatty acid desaturase (FADS2) deficiency. EMBO Rep. 15: 110–120. 24378641 10.1002/embr.201338041 PMC4303455

[19] Kilkenny C., Browne W. J., Cuthill I. C., Emerson M., and Altman D. G. 2010 Improving bioscience research reporting: the ARRIVE guidelines for reporting animal research. J. Pharmacol. Pharmacother. 1: 94–99. 21350617 10.4103/0976-500X.72351 PMC3043335

[20] Stoffel W. 1965 Chemical synthesis of H3- and 1-C-14-labeled polyunsaturated fatty acids. J. Am. Oil Chem. Soc. 42: 583–587. 14328356 10.1007/BF02541294

[21] Williams J., Wood J., Pandarinathan L., Karanian D. A., Bahr B. A., Vouros P., and Makriyannis A. 2007 Quantitative method for the profiling of the endocannabinoid metabolome by LC-atmospheric pressure chemical ionization-MS. Anal. Chem. 79: 5582–5593. 17600384 10.1021/ac0624086

[22] Nagy A., Gertsenstein M., Vintersten K., and Behringer R. 2006 Preparing mouse embryo fibroblasts. CSH Protoc. 2006: . 10.1101/pdb.prot4398 22485758

[23] Walter A., Saric T., Hescheler J., and Papadopoulos S. 2016 Calcium imaging in pluripotent stem cell-derived cardiac myocytes. Methods Mol. Biol. 1353: 131–146. 26025623 10.1007/7651_2015_267

[24] Bannon A. W., and Malmberg A. B. 2007 Models of nociception: hot-plate, tail-flick, and formalin tests in rodents. Curr. Protoc. Neurosci. Chapter 8: Unit 8.9. 10.1002/0471142301.ns0809s41 18428666

[25] Christie W. W. 1998 Gas chromatography-mass spectrometry methods for structural analysis of fatty acids. Lipids. 33: 343–353. 9590621 10.1007/s11745-998-0214-x

[26] Park W. J., Kothapalli K. S., Lawrence P., Tyburczy C., and Brenna J. T. 2009 An alternate pathway to long-chain polyunsaturates: the FADS2 gene product Delta8-desaturates 20:2n-6 and 20:3n-3. J. Lipid Res. 50: 1195–1202. 19202133 10.1194/jlr.M800630-JLR200 PMC2681401

[27] Mead J. F., and Slaton W. H. Jr. 1956 Metabolism of essential fatty acids. III. Isolation of 5,8,11-eicosatrienoic acid from fat-deficient rats. J. Biol. Chem. 219: 705–709. 13319291

[28] Priller J., Briley E. M., Mansouri J., Devane W. A., Mackie K., and Felder C. C. 1995 Mead ethanolamide, a novel eicosanoid, is an agonist for the central (CB1) and peripheral (CB2) cannabinoid receptors. Mol. Pharmacol. 48: 288–292. 7651362

[29] Sugiura T., Kondo S., Kishimoto S., Miyashita T., Nakane S., Kodaka T., Suhara Y., Takayama H., and Waku K. 2000 Evidence that 2-arachidonoylglycerol but not N-palmitoylethanolamine or anandamide is the physiological ligand for the cannabinoid CB2 receptor. Comparison of the agonistic activities of various cannabinoid receptor ligands in HL-60 cells. J. Biol. Chem. 275: 605–612. 10617657 10.1074/jbc.275.1.605

[30] Shao Z., Yin J., Chapman K., Grzemska M., Clark L., Wang J., and Rosenbaum D. M. 2016 High-resolution crystal structure of the human CB1 cannabinoid receptor. Nature. 540: 602–606. 27851727 10.1038/nature20613 PMC5433929

[31] Hua T., Vemuri K., Nikas S. P., Laprairie R. B., Wu Y., Qu L., Pu M., Korde A., Jiang S., Ho J. H., 2017 Crystal structures of agonist-bound human cannabinoid receptor CB1. Nature. 547: 468–471. 28678776 10.1038/nature23272 PMC5793864

[32] Liu Q. R., Huang N. S., Qu H., O’Connell J. F., Gonzalez-Mariscal I., Santa-Cruz-Calvo S., Doyle M. E., Xi Z. X., Wang Y., Onaivi E. S., and Egan J. M. 2019 Identification of novel mouse and rat CB1R isoforms and in silico modeling of human CB1R for peripheral cannabinoid therapeutics. Acta Pharmacol. Sin. 40: 387–397. 30202012 10.1038/s41401-018-0152-1 PMC6460360

[33] Bradbury J. 2011 Docosahexaenoic acid (DHA): an ancient nutrient for the modern human brain. Nutrients. 3: 529–554. 22254110 10.3390/nu3050529 PMC3257695

[34] Wood J. T., Williams J. S., Pandarinathan L., Janero D. R., Lammi-Keefe C. J., and Makriyannis A. 2010 Dietary docosahexaenoic acid supplementation alters select physiological endocannabinoid-system metabolites in brain and plasma. J. Lipid Res. 51: 1416–1423. 20071693 10.1194/jlr.M002436 PMC3035504

[35] Gao X. B., and Horvath T. L. 2016 Feeding behavior: hypocretin/orexin neurons act between food seeking and eating. Curr. Biol. 26: R845–R847. 27676302 10.1016/j.cub.2016.07.069

[36] Berrendero F., Flores A., and Robledo P. 2018 When orexins meet cannabinoids: Bidirectional functional interactions. Biochem. Pharmacol. 157: 43–50. 30171834 10.1016/j.bcp.2018.08.040

[37] Re G., Barbero R., Miolo A., and Di Marzo V. 2007 Palmitoyle­thanolamide, endocannabinoids and related cannabimimetic compounds in protection against tissue inflammation and pain: potential use in companion animals. Vet. J. 173: 21–30. 16324856 10.1016/j.tvjl.2005.10.003

[38] Romero T. R., and Duarte I. D. 2012 N-palmitoyl-ethanolamine (PEA) induces peripheral antinociceptive effect by ATP-sensitive K+-channel activation. J. Pharmacol. Sci. 118: 156–160. 22343363 10.1254/jphs.11150fp

[39] Artmann A., Petersen G., Hellgren L. I., Boberg J., Skonberg C., Nellemann C., Hansen S. H., and Hansen H. S. 2008 Influence of dietary fatty acids on endocannabinoid and N-acylethanolamine levels in rat brain, liver and small intestine. Biochim. Biophys. Acta. 1781: 200–212. 18316044 10.1016/j.bbalip.2008.01.006

[40] Watanabe S., Doshi M., and Hamazaki T. 2003 n-3 Polyunsaturated fatty acid (PUFA) deficiency elevates and n-3 PUFA enrichment reduces brain 2-arachidonoylglycerol level in mice. Prostaglandins Leukot. Essent. Fatty Acids. 69: 51–59. 12878451 10.1016/s0952-3278(03)00056-5

[41] Burston J. J., and Woodhams S. G. 2014 Endocannabinoid system and pain: an introduction. Proc. Nutr. Soc. 73: 106–117. 24148358 10.1017/S0029665113003650

[42] Cravatt B. F., and Lichtman A. H. 2004 The endogenous cannabinoid system and its role in nociceptive behavior. J. Neurobiol. 61: 149–160. 15362158 10.1002/neu.20080

[43] Schreiber A. K., Neufeld M., Jesus C. H., and Cunha J. M. 2012 Peripheral antinociceptive effect of anandamide and drugs that affect the endocannabinoid system on the formalin test in normal and streptozotocin-diabetic rats. Neuropharmacology. 63: 1286–1297. 22959964 10.1016/j.neuropharm.2012.08.009

[44] Rapoport S. I. 2008 Brain arachidonic and docosahexaenoic acid cascades are selectively altered by drugs, diet and disease. Prostaglandins Leukot. Essent. Fatty Acids. 79: 153–156. 18973997 10.1016/j.plefa.2008.09.010 PMC4576349

[45] Batetta B., Griinari M., Carta G., Murru E., Ligresti A., Cordeddu L., Giordano E., Sanna F., Bisogno T., Uda S., 2009 Endocannabinoids may mediate the ability of (n-3) fatty acids to reduce ectopic fat and inflammatory mediators in obese Zucker rats. J. Nutr. 139: 1495–1501. 19549757 10.3945/jn.109.104844

[46] Brenner R. R., and Peluffo R. O. 1969 Regulation of unsaturated fatty acids biosynthesis. I. Effect of unsaturated fatty acid of 18 carbons on the microsomal desaturation of linoleic acid into gamma-linolenic acid. Biochim. Biophys. Acta. 176: 471–479. 5800038 10.1016/0005-2760(69)90214-8

[47] Tu W. C., Cook-Johnson R. J., James M. J., Muhlhausler B. S., and Gibson R. A. 2010 Omega-3 long chain fatty acid synthesis is regulated more by substrate levels than gene expression. Prostaglandins Leukot. Essent. Fatty Acids. 83: 61–68. 20573490 10.1016/j.plefa.2010.04.001

[48] Lands W. E., Inoue M., Sugiura Y., and Okuyama H. 1982 Selective incorporation of polyunsaturated fatty acids into phosphatidylcholine by rat liver microsomes. J. Biol. Chem. 257: 14968–14972. 7174678

[49] Maurelli S., Bisogno T., De Petrocellis L., Di Luccia A., Marino G., and Di Marzo V. 1995 Two novel classes of neuroactive fatty acid amides are substrates for mouse neuroblastoma ‘anandamide amidohydrolase’. FEBS Lett. 377: 82–86. 8543025 10.1016/0014-5793(95)01311-3

[50] Maccarrone M., van der Stelt M., Rossi A., Veldink G. A., Vliegenthart J. F., and Agro A. F. 1998 Anandamide hydrolysis by human cells in culture and brain. J. Biol. Chem. 273: 32332–32339. 9822713 10.1074/jbc.273.48.32332

[51] de Urquiza A. M., Liu S., Sjoberg M., Zetterstrom R. H., Griffiths W., Sjovall J., and Perlmann T. 2000 Docosahexaenoic acid, a ligand for the retinoid X receptor in mouse brain. Science. 290: 2140–2144. 11118147 10.1126/science.290.5499.2140

[52] Lengqvist J., Mata De Urquiza A., Bergman A. C., Willson T. M., Sjovall J., Perlmann T., and Griffiths W. J. 2004 Polyunsaturated fatty acids including docosahexaenoic and arachidonic acid bind to the retinoid X receptor alpha ligand-binding domain. Mol. Cell. Proteomics. 3: 692–703. 15073272 10.1074/mcp.M400003-MCP200

[53] Kliewer S. A., Sundseth S. S., Jones S. A., Brown P. J., Wisely G. B., Koble C. S., Devchand P., Wahli W., Willson T. M., Lenhard J. M., 1997 Fatty acids and eicosanoids regulate gene expression through direct interactions with peroxisome proliferator-activated receptors alpha and gamma. Proc. Natl. Acad. Sci. USA. 94: 4318–4323. 9113987 10.1073/pnas.94.9.4318 PMC20720

[54] Evans R. M., and Mangelsdorf D. J. 2014 Nuclear receptors, RXR, and the big bang. Cell. 157: 255–266. 24679540 10.1016/j.cell.2014.03.012 PMC4029515

