# Supplementary material for: Novel CB1-ligands maintain homeostasis of the endocannabinoid system in ω3- and ω6-long-chain-PUFA deficiency
Source: J Lipid Res. 2019 Jun 5;60(8):1396–409. doi: 10.1194/jlr.M094664 (PMC6672042; doi:10.1194/jlr.M094664)
Supplement: Supplemental Data [file supp_60_8_1396__index.html]

Novel CB1-ligands maintain homeostasis of the endocannabinoid system in ω3- and ω6-long-chain-PUFA deficiency — Supplemental Data 

# Novel CB1-ligands maintain homeostasis of the endocannabinoid system in ω3- and ω6-long-chain-PUFA deficiency

## Supplemental Data

- Supplemental Figures
