# Supplementary material for: Novel CB1-ligands maintain homeostasis of the endocannabinoid system in ω3- and ω6-long-chain-PUFA deficiency
Source: J Lipid Res. 2019 Jun 5;60(8):1396–409. doi: 10.1194/jlr.M094664 (PMC6672042; doi:10.1194/jlr.M094664)
Supplement: Supplemental Data [file supp_M094664_Supplemental-Figures.docx]

**Supplemental Information**

**Novel CB1-ligands maintain homeostasis of the endocannabinoid-system in ω3- and ω6- long chain-PUFA deficiency**

**Ina Hammels*,**, Erika Binczek*, Inga Schmidt-Soltau*, Britta Jenke*, Andreas Thomas†, Matthias Vogel† , Mario Thevis†, Dilyana Filipova††, Symeon Papadopoulos†† and Wilhelm Stoffel*,****^[[1]](#footnote-1)^

*** Center of Molecular Medicine (CMMC), Laboratory of Molecular Neurosciences, Institute of Biochemistry, University of Cologne, Joseph-Stelzmann-Strasse 52, 50931 Cologne, Germany**

**** CECAD (Cluster of Excellence, Cellular Stress Response in Aging Related Diseases), University of Cologne, 50931 Cologne, Germany**

**† Institute of Biochemistry, Deutsche Sporthochschule (DSHS) Cologne, 50933 Cologne, Germany**

**††** Institute of Vegetative Physiology, **Center of Physiology and Pathophysiology, University of Cologne, 50931 Cologne, Germany**

**
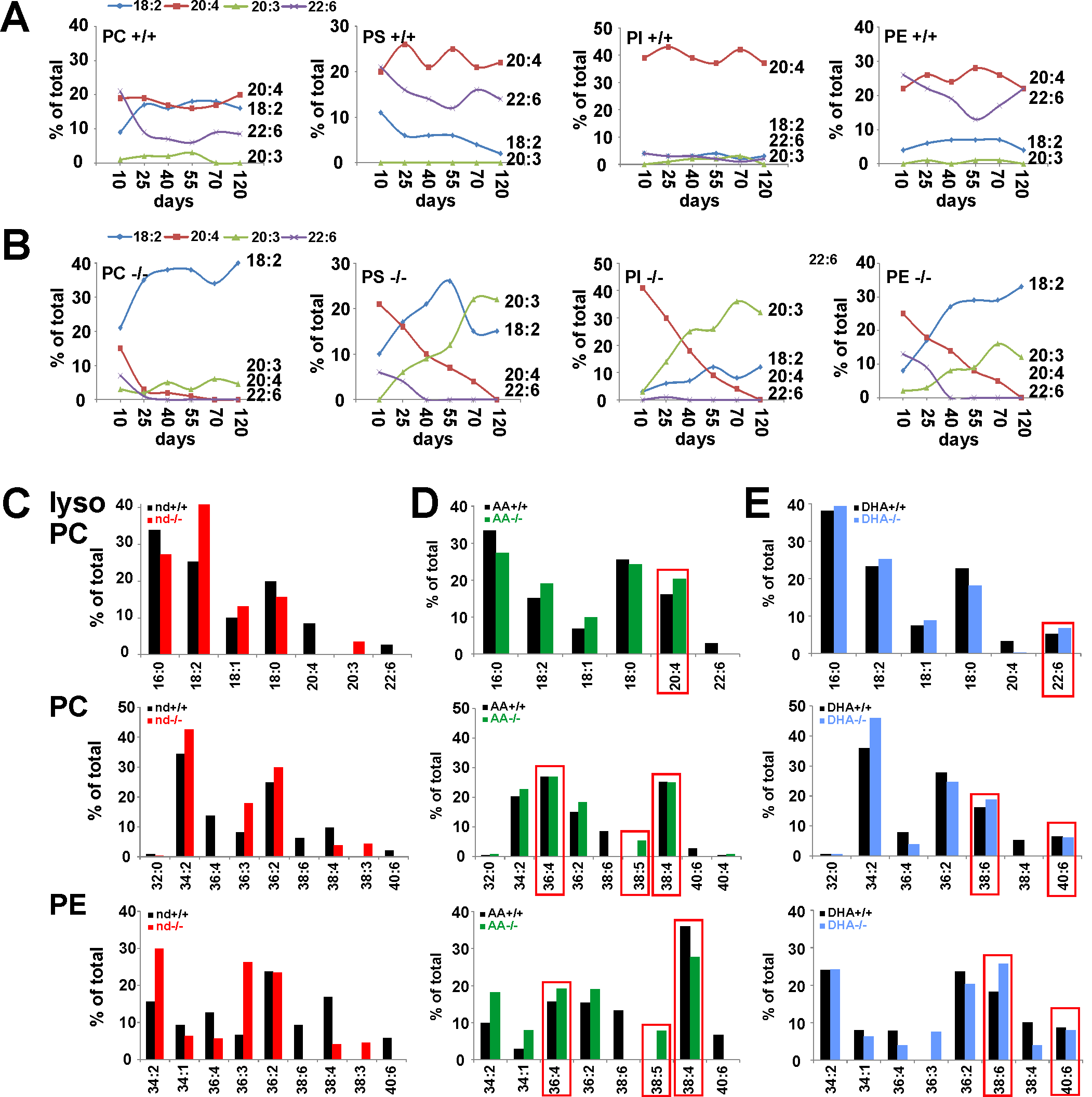
**

**Supplemental Figure S1 Analysis of the phospholipidomes of liver and serum, and kinetics of the PUFA pattern of liver.** A, B: GC/MS analysis of the dynamics of the PUFA-pattern of liver-phospholipids of WT and *fads2-/-* mice on normal diet (nd). PUFA substitution of phospholipid-classes PC, PS, PI and PE in liver of (A) WT and (B) *fads2-/-* mice. Tracings: 18:2, 20:3, 20:4 and 22:6 at 10, 25, 40, 55, 70 and 120 days. C-E: MS/MS analysis of lipid-classes of serum of cohorts of WT and *fads2-/-* (4mo) mice on (C) normal diet (nd) (WT: black bars, *fads2-/-*: red bars), (D) ω6-AA-diet (WT: black bars, *fads2-/-*: green bars) and (E) ω3-DHA-diet (WT: black bars, *fads2-/-*: blue bars). A pool of n=3 per genotype was used.


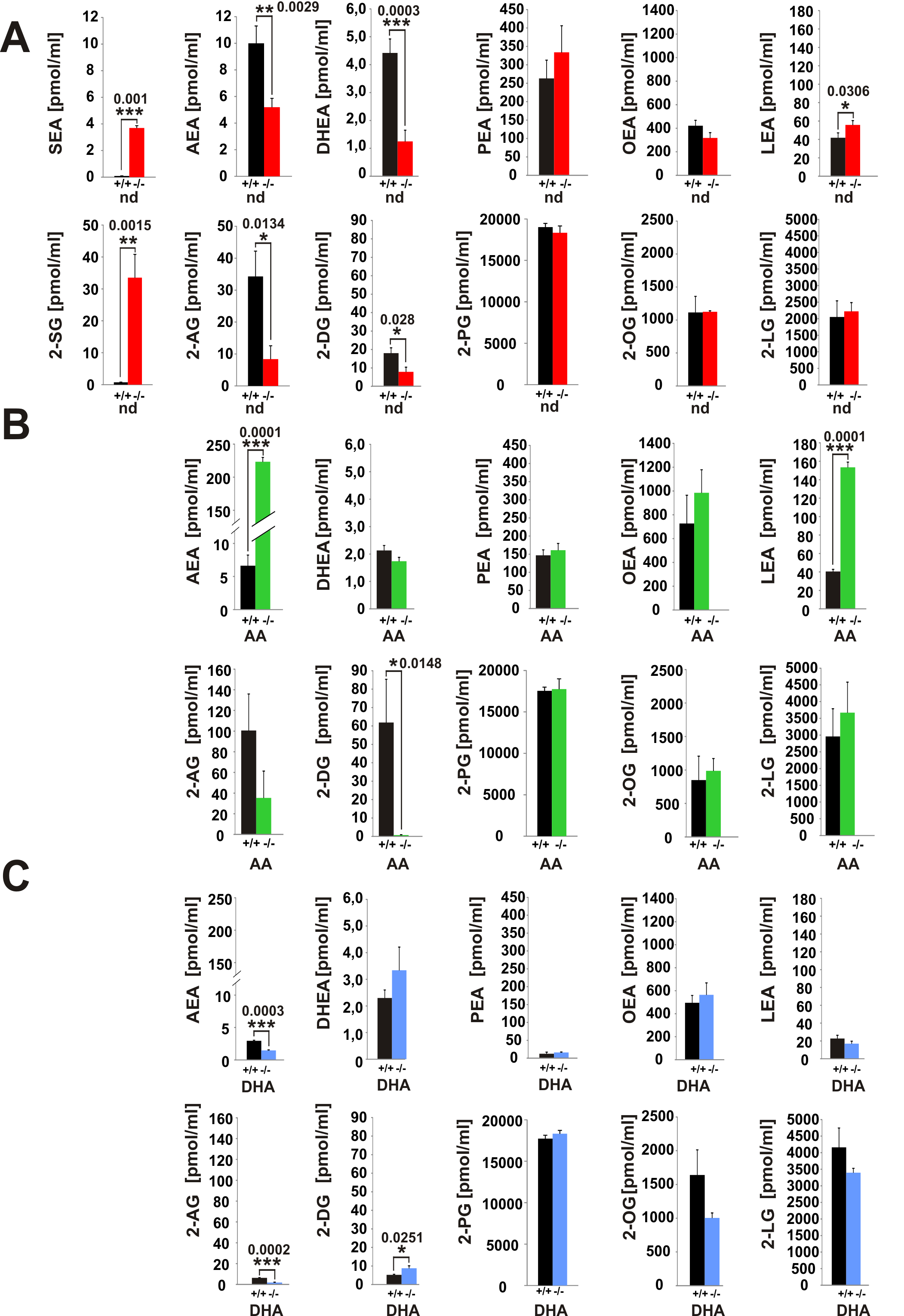


**Supplemental Figure S2:** **Novel endocannabinoids 20:3^5,11,14^-(sciadonoyl)-ethanolamide (SEA) and 2-20:3^5,11,14^-(sciadonoyl)-glycerol (2-SG) in the endocannabinoid pattern of serum of nd-*fads2-/-* mice.** A-C: Separation and quantification of endocannabinoids in lipid extracts of (A) nd- (WT: black bars, *fads2-/-*: red bars), (B) ω6-AA- (WT: black bars, *fads2-/-*: green bars) and (C) ω3-DHA-WT and -*fads2-/-* mice (4mo) (WT: black bars, *fads2-/-*: blue bars) by HPLC-MS/MS. Data represent mean±SEM. Two-tailed Student’s t-test, p-values ≤ 0.05 *, ≤ 0.01 **, ≤ 0.001 *** were considered significant. N=8 per genotype. Sciadonoyl-ethanolamide (SEA), 2-sciadonoyl-glycerol (2-SG), palmitoyl-ethanolamide (PEA), oleoyl-ethanolamide (OEA), 2-oleoyl-glycerol (2-OG), linoyl-ethanolamide (LEA), 2-linoyl-glycerol (2-LG), arachidonoyl-ethanolamide (AEA), 2-arachidonoyl-glycerol (2-AG), docosahexanoyl-ethanolamide (DHEA), 2-docosahexanoyl-glycerol (2-DG)


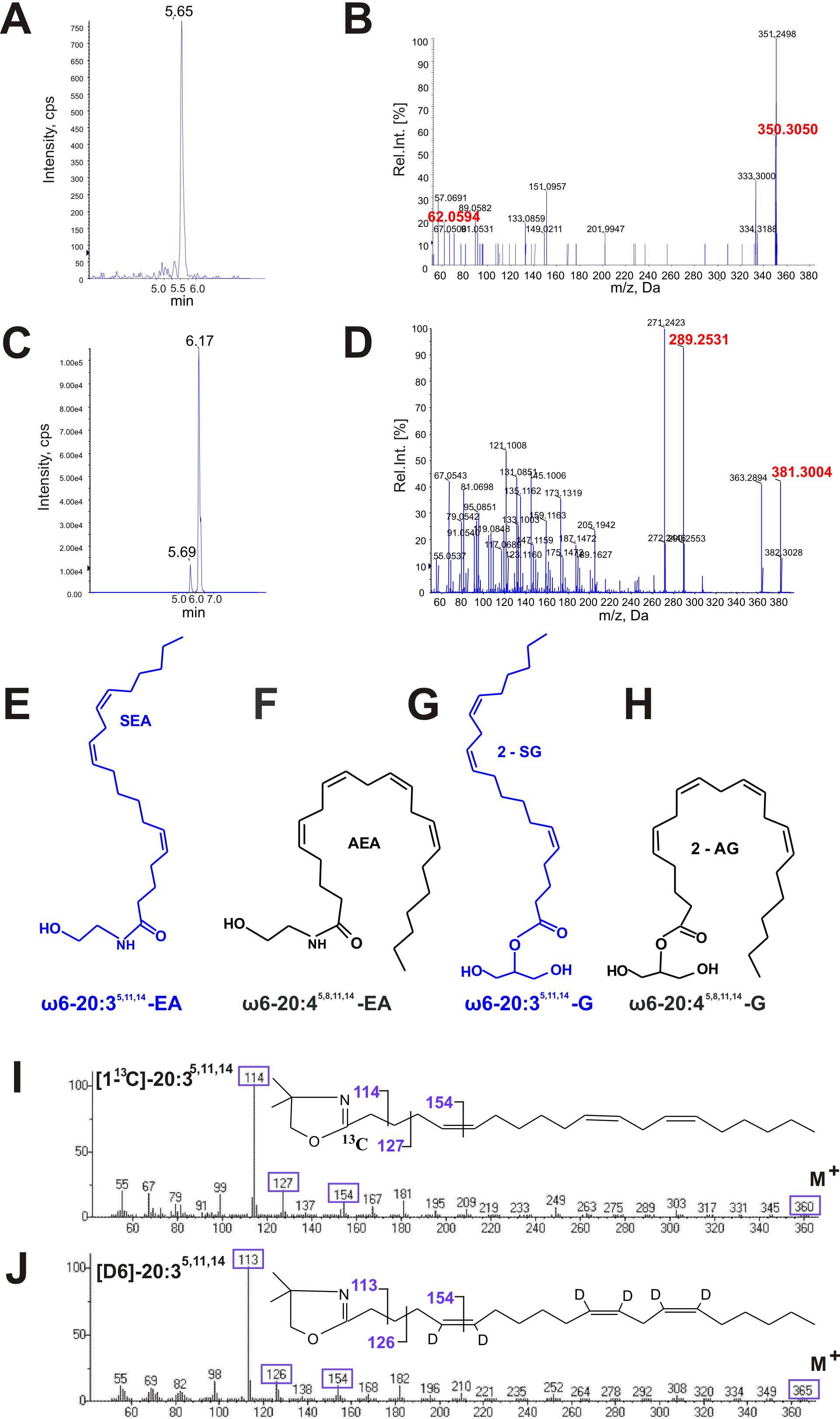


**Supplemental Figure S3:** **Structural analysis of novel endocannabinoids isolated from brain of nd-*fads2-/-* mice.** A-J: LC-MS/MS of extracted ion chromatograms (XIC) (A, B) of SEA, transition 350.3-62.06 Da, and TOF product 350.3 at 5.6099 to 5.6831 min; (C, D) 2-SG, transition 381.3-289.3 Da, and TOF product 381.3 at 6.1362 to 6.2217 min. Structures of (E) ω6-20:3^5,11,14^-EA, (F) ω6-20:4^5,8,11,14^-EA, (G) ω6-20:3^5,11,14^-G and (H) ω6-20:4^5,8,11,14^-G. Characterization of synthetic (I) [1-^13^C]- and (J) [D6]-labeled ω6-20:3^5,11,14^.

The chemical structures and analytical data of the novel endocannabinoids, isolated and analyzed by LC-MS/MS from nd-*fads2-/-* mice (Supplemental Figure S3A-D), proved identical with that of the synthetic unlabeled and [1-C^13^]- and [D6]-labelled ω6-20:3^5,11,14^-EA and ω6-20:3^5,11,14^-G (Supplemental Figure S3E-F).


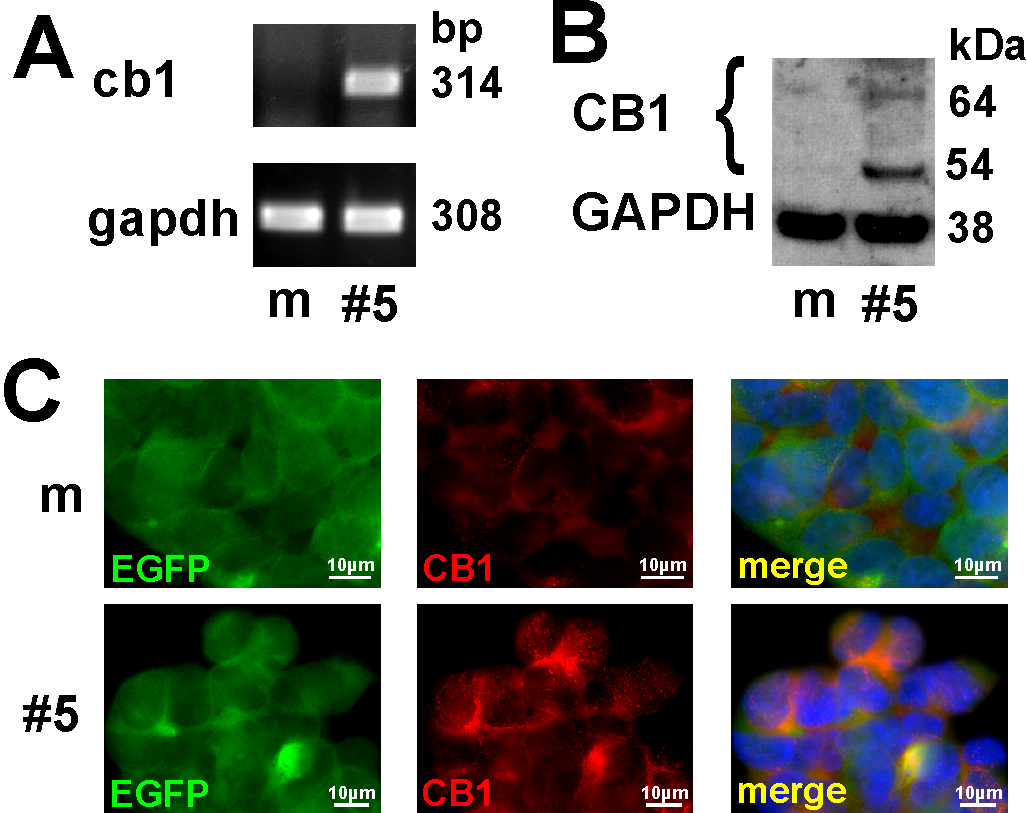


**Supplemental Figure S4: Characterization of the mock and *cb1*-transfected HEK293-cells**. A: PCR of *cannabinoid receptor 1* (*cb1*) and *glycerinaldehyd-3-phosphat-dehydrogenase (gapdh)* of cRNA, B: Western blot of anti-CB1 and anti-GAPDH of protein lysates and C: IHC localization of CB1 and EGFP of mock (m) and *cb1*-transfected (clone #5) HEK293-cells.

1. To whom correspondence should be addressed. E-mail: wilhelm.stoffel@uni-koeln.de [↑](#footnote-ref-1)
